# Phospholipid synthesis fueled by lipid droplets drives the structural development of poliovirus replication organelles

**DOI:** 10.1371/journal.ppat.1007280

**Published:** 2018-08-27

**Authors:** Ekaterina G. Viktorova, Jules A. Nchoutmboube, Lauren A. Ford-Siltz, Ethan Iverson, George A. Belov

**Affiliations:** 1 Department of Veterinary Medicine and Virginia-Maryland College of Veterinary Medicine, University of Maryland, College Park, MD, United States of America; 2 Department of Molecular and Cellular Biology, University of Maryland, College Park, MD, United States of America; University of Heidelberg, GERMANY

## Abstract

Rapid development of complex membranous replication structures is a hallmark of picornavirus infections. However, neither the mechanisms underlying such dramatic reorganization of the cellular membrane architecture, nor the specific role of these membranes in the viral life cycle are sufficiently understood. Here we demonstrate that the cellular enzyme CCTα, responsible for the rate-limiting step in phosphatidylcholine synthesis, translocates from the nuclei to the cytoplasm upon infection and associates with the replication membranes, resulting in the rerouting of lipid synthesis from predominantly neutral lipids to phospholipids. The bulk supply of long chain fatty acids necessary to support the activated phospholipid synthesis in infected cells is provided by the hydrolysis of neutral lipids stored in lipid droplets. Such activation of phospholipid synthesis drives the massive membrane remodeling in infected cells. We also show that complex membranous scaffold of replication organelles is not essential for viral RNA replication but is required for protection of virus propagation from the cellular anti-viral response, especially during multi-cycle replication conditions. Inhibition of infection-specific phospholipid synthesis provides a new paradigm for controlling infection not by suppressing viral replication but by making it more visible to the immune system.

## Author summary

The cellular pathways hijacked to support viral replication may provide a promising class of targets for anti-viral therapeutics, which will be effective against broad groups of viruses relying on the same cellular pathways, and will likely be refractory to the development of resistance since cellular factors are not subject to selection. All (+)RNA viruses share the requirement for cellular membranes to assemble replication complexes. Here we investigated the mechanism underlying the massive membrane remodeling in poliovirus-infected cells. Our results demonstrate reorganization of the cellular lipid synthesizing machinery upon infection and identify lipid droplets as the organelles supporting the structural development of the replication membranes. Moreover, we show that inhibition of the infection-specific phospholipid synthesis renders virus propagation much more vulnerable to the cellular anti-viral defenses, providing a new direction for the development of anti-viral therapeutics.

## Introduction

The positive strand RNA ((+)RNA) viruses of eukaryotes universally assemble their RNA replication machinery in association with specialized membranous domains, featuring unique lipid and protein composition [1–3]. It is hypothesized that membranes may facilitate replication by increasing local concentration of the viral proteins, providing a scaffold for assembly of the multi-subunit replication complexes, and/or by hiding the dsRNA replication intermediates from cellular sensors of infection [4]. To trick the infected cells into building new membranous structures, viruses have to reorganize the complex network of cellular pathways controlling lipid synthesis, catabolism and membrane trafficking. Yet, in spite of the central role of the membranous replication organelles in the life cycle of (+)RNA viruses, our knowledge about the mechanistic details of their formation in most viral systems is very limited, and the experimental evidence supporting their importance for specific replication steps is scarce.

*Picornavidae* is a family of small non-enveloped (+)RNA viruses of vertebrate hosts, and the number and diversity of known picornaviruses is rapidly increasing. Among picornaviruses are important human and animal pathogens such as poliovirus, etiological agent of poliomyelitis; Coxsackie viruses, associated with type I diabetes, myocarditis, and dilated cardiomyopathy; rhinoviruses, the main cause of the common cold; foot and mouth disease virus, the major concern for animal husbandry worldwide; and others [5–9]. Poliovirus, a representative of the *Enterovirus* genus, is the prototype member of the *Picornaviridae* family. Its genome RNA of ~7500 nt is directly translated in a cap-independent manner into one polyprotein (~200 KDa) which undergoes a cascade of proteolytic cleavages generating a dozen of mature peptides and intermediate cleavage products. Proteins encoded in the P2-P3 region of the viral genome as well as the corresponding cleavage intermediates are responsible for genome replication, while the P1 region codes for capsid proteins.

Poliovirus infection results in rapid dramatic reorganization of the cellular membrane architecture. Within a few hours post infection, new membranous structures harboring the viral replication complexes fill the cytoplasm. The 3D reconstruction of picornavirus replication organelles show that during the active RNA replication stage of infection, they are formed by tightly associated convoluted single-walled membranous compartments which later undergo transition into double-membrane vesicles [10–12]. Electron microscopy studies of cells infected with diverse picornaviruses demonstrate the virtually identical appearance of the replication membranes, at least at certain stages of infection, strongly suggesting a common mechanism behind their formation [13, 14]. Based on the superficial morphological features of the replication membranes observed in thin section electron microscopy images, it was previously proposed that hijacking the normal cellular membrane metabolism pathways such as the secretory pathway and/or autophagy could be responsible for the membrane remodeling in infected cells. However, the accumulated evidence argue against such straightforward interpretation. First, picornaviruses vary greatly in their sensitivity to pharmacological or genetic manipulations of these pathways [13, 15, 16]. Second, it was recognized that the elements of the secretory pathway are involved in imparting the unique characteristics of the replication membranes such as their enrichment in phosphatidylinositol-4 phosphate and activated small GTPases of the Arf family, rather than in the structural development of these replication platforms [17–20]. Third, the activation of autophagy appears important for the maturation of infectious virions, but not for RNA replication, at least for poliovirus and related viruses [15, 21, 22].

It was previously established that in cells infected with poliovirus and encephalomyocarditis virus, a picornavirus from the *Cardiovirus* genus, synthesis of phospholipids is activated, and the newly-synthesized phospholipid molecules are found in the membrane fraction associated with the viral RNA-dependent RNA polymerase activity, suggesting that they may contribute to the development of the replication membranes [23–25]. However, the significance and the mechanisms of the infection-specific activation of phospholipid synthesis remained unknown. We previously demonstrated that in picornavirus-infected cells, long chain acyl-CoA synthetase activity is rapidly upregulated and is associated with the highly increased rate of long chain fatty acid (FA) import and their re-routing from triglyceride (TG) to phosphatidylcholine (PC) synthesis. Similar changes in long chain FA metabolism were observed in different cell types infected with diverse picornaviruses, indicating that they constitute a universal attribute of picornavirus infection [26].

Here, we further investigated the mechanism of infection-specific activation of phospholipid synthesis and its role in the development of the viral replication organelles. We demonstrate that activation of lipolysis of neutral lipids in lipid droplets, but not import or *de novo* synthesis, supplies the bulk of long chain FAs for infection-specific phospholipid production. We show that the key enzyme in phosphatidylcholine synthesis, CTP-phosphocholine-cytidyl transferase alpha (CCTα) translocates from the nuclei of infected cells and associates with membranes of the viral replication complexes. Inhibition of PC synthesis disrupts the normal structural development of the replication organelles. In the absence of a tight membranous matrix, the first round of viral replication can proceed normally, but viral propagation in multiple rounds of infection is severely compromised. Cellular sensors of infection are activated stronger, and viral replication becomes more sensitive to the anti-viral response if synthesis of structural phospholipids is inhibited. Thus, our research establishes an important role of lipid droplets in picornavirus infection and provides a novel paradigm for controlling viral infections not by targeting the viral replication *per se* but by making it more visible to the host defense mechanisms.

## Results

### Overexpression of CCTα reroutes the metabolic flux of long chain FAs from neutral to membrane lipid synthesis, similar to the phenotype observed in infected cells

**Fig 1:**
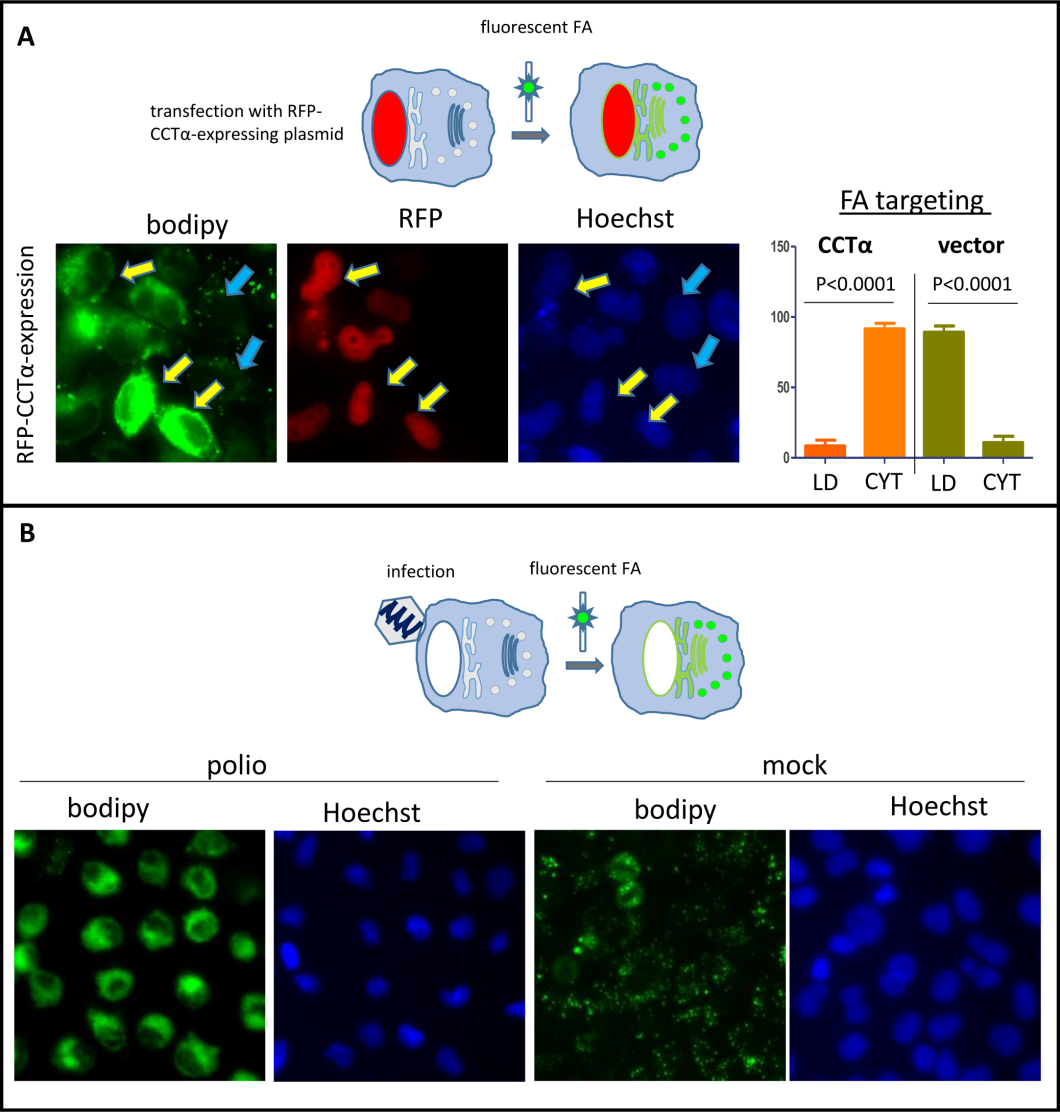
Overexpression of CCTα phenotypically recapitulates retargeting of long chain FAs from neutral to membrane lipid synthesis observed in poliovirus-infected cells. **A.** HeLa cells were transfected with a CCTα-RFP expressing plasmid, and the next day they were incubated for 1 h with Bodipy C4/C9 in the medium. Yellow arrows indicate cells expressing the fusion protein, blue arrows show non-transfected cells. **B.** HeLa cells were infected (mock-infected) with poliovirus at an MOI of 10 PFU/cell, and at 4 h p.i. they were incubated with Bodipy C4/C9 in the medium for 1 h.

We previously showed that infection-specific activation of phospholipid synthesis does not depend on transcription of cellular genes [27]. Together with the rapid shut-off of cellular mRNA translation in poliovirus-infected cells [28], this suggests that the activation of phospholipid synthesis must depend on post-translational regulation of the cellular enzymes already present before infection. Earlier studies of activation of PC synthesis in poliovirus-infected cells proposed that the rate limiting reaction activated upon infection is the synthesis of CDP-choline, a substrate for phosphocholine head group transfer to diacylglycerol; however, the mechanism of such activation has not been established [29]. The human genome contains two genes coding for CTP-phosphocholine-cytidyl transferase (CCT) enzymes, which can synthesize CDP-choline. CCTα is ubiquitously expressed, while CCTβ has a restricted tissue-specific expression pattern [30, 31]. Due to the presence of N-terminal nuclear localization signal, CCTα is partitioned between the nuclear depot of inactive enzyme and the cytoplasmic pool containing the activated form [32, 33]. To directly observe if CCTα activity can guide the changes in lipid metabolism similar to those found upon infection, we overexpressed a CCTα fused with a red fluorescent protein (CCTα-RFP) in HeLa cells and monitored incorporation of a fluorescent long chain FA analog Bodipy C4/C9, into cellular structures. Bodipy C4/C9 mimics long chain FAs with C18 backbone and can be either incorporated in neutral lipids and stored in lipid droplets, or metabolized into membrane phospholipids. We previously validated it as a convenient tool to study membrane synthesis in infected cells [26, 34]. HeLa cells were transfected with a CCTα-RFP-expressing plasmid and the next day, Bodipy C4/C9 was added to the incubation medium for 1h (Fig 1A). For direct comparison, HeLa cells were infected with poliovirus at an MOI of 10 and Bodipy C4/C9 was added to the incubation medium of infected cells for 1 h at 4 h p.i. (Fig 1B), similar to the experiments described in [26, 34]. Microscopic examination of cells revealed that most of the RFP-CCTα signal was concentrated in the nuclei, as was previously described for CCTα [35], thus the fusion recapitulated behavior of the native protein. Even if the bulk of overexpressed CCTα was confined in the nuclei, this was sufficient to significantly increase the activity of the enzyme because the incorporation of Bodipy C4/C9 was much stronger in cells expressing CCTα-RFP (Fig 1A, yellow arrows), and in those cells the signal of the fluorescent FA was distributed in the cytoplasm, reflecting its partitioning to the membrane phospholipids. At the same time, in cells that did not express CCTα-RFP, the signal of Bodipy C4/C9 was found in lipid droplets (Fig 1A, blue arrows). Similarly, in mock-infected cells, Bodipy C4/C9 signal was localized in lipid droplets, but in infected cells the incorporation of the fluorescent FA was much stronger, and it was redistributed in the cytoplasmic membranes (compare Fig 1B mock- and polio-infected cells). Thus, overexpression of CCTα phenotypically recapitulates changes in the long chain FA metabolism observed during poliovirus infection.

### CCTα translocates from the nuclei of infected cells and this translocation requires activity of the viral protease 2A

**Fig 2:**
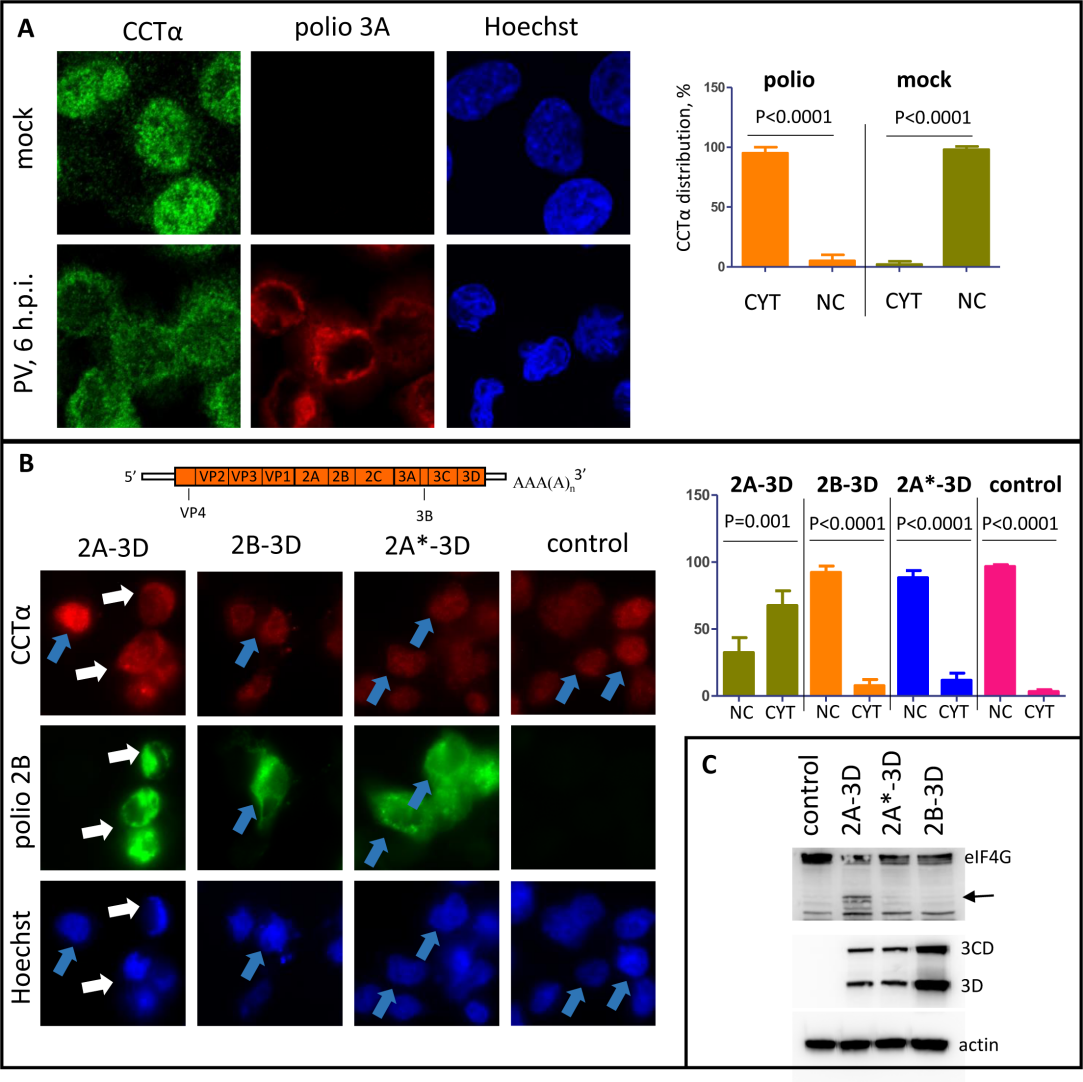
CCTα translocates from the nuclei to the cytoplasm in infected cells. **A.** Translocation of CCTα into the cytoplasm upon infection. Confocal images of HeLa cells infected with poliovirus at an MOI of 10 PFU/cell, fixed, and processed for imaging of endogenous CCTα at 4 hp.i. Replication complexes are visualized by staining for a viral antigen 3A. **B.** Translocation of CCTα upon replication-independent expression of the fragments of poliovirus polyprotein. HeLa cells were transfected with plasmids coding for the indicated polyprotein fragments under control of T7 promotor and infected with a vaccinia virus expressing T7 RNA polymerase. Control cells were transfected with an empty vector. The next day, the cells were fixed and processed for imaging of endogenous CCTα and viral antigen 2B. **C.** Expression of the whole viral polyprotein fragments in the samples produced as in (B) is confirmed by western blot with antibodies against the viral protein 3D encoded in the very 3’-terminal part of the genome. The lack of 2A proteolytic activity upon expression of the fragment with mutant 2A*-3D and 2B-3D is confirmed by the lack of processing of eIF4G (arrow). Actin is shown as a loading control.

Next, we investigated what happens to the endogenous CCTα upon poliovirus infection. Immunostaining revealed that in mock-infected HeLa cells, the enzyme was mostly localized in the nuclei, with some scattered spots around the cytoplasm (Fig 2A). In poliovirus-infected cells, however, nuclear CCTα signal was weaker than that in the cytoplasm, showing that the enzyme is translocated from the nucleus and associates with some cytoplasmic structures (Fig 2A). CCTα staining in infected cells occupied the same cellular area as the viral replication organelles, visualized by staining of a viral antigen 3A, although there was no direct co-localization between CCTα and the viral protein(s) (Fig 2A).

To understand what viral proteins are responsible for CCTα translocation we expressed fragments of the poliovirus polyprotein in a replication-independent manner using a vaccinia-T7 expression system [36]. In HeLa cells expressing poliovirus proteins from 2A to 3D, we observed a significant translocation of CCTα from the nuclei, similar to that in infected cells (Fig 2B, white arrows). In cells expressing poliovirus proteins from 2B to 3D, CCTα localization was almost exclusively nuclear, similar to that in cells transfected with an empty vector (Fig 2B, blue arrows), even though the 2B-3D construct was expressed at a somewhat higher level than 2A-3D (Fig 2C). 2A is a protease and it was previously shown to be responsible for disruption of the barrier function of nuclear envelope [37, 38]. To see if the protease activity of 2A is important for CCTα translocation, we expressed a 2A*-3D polyprotein fragment with 2A containing a mutation in the catalytic triad. Both 2A-3D and 2A*-3D constructs were expressed at a similar level, and the lack of catalytic activity of 2A* was confirmed by the inhibition of cleavage of the cellular translation initiation factor eIF-4G, a well-known 2A-dependent process (Fig 2C). Like in the cells expressing 2B-3D polyprotein fragment, CCTα was almost exclusively confined to the nuclei if 2A was inactivated (Fig 2B). Thus, poliovirus infection induces massive translocation of CCTα from the nucleus to the cytoplasm and this process requires proteolytic activity of 2A.

### CCTα associates with the replication membranes and controls activation of phospholipid synthesis in infected cell

**Fig 3:**
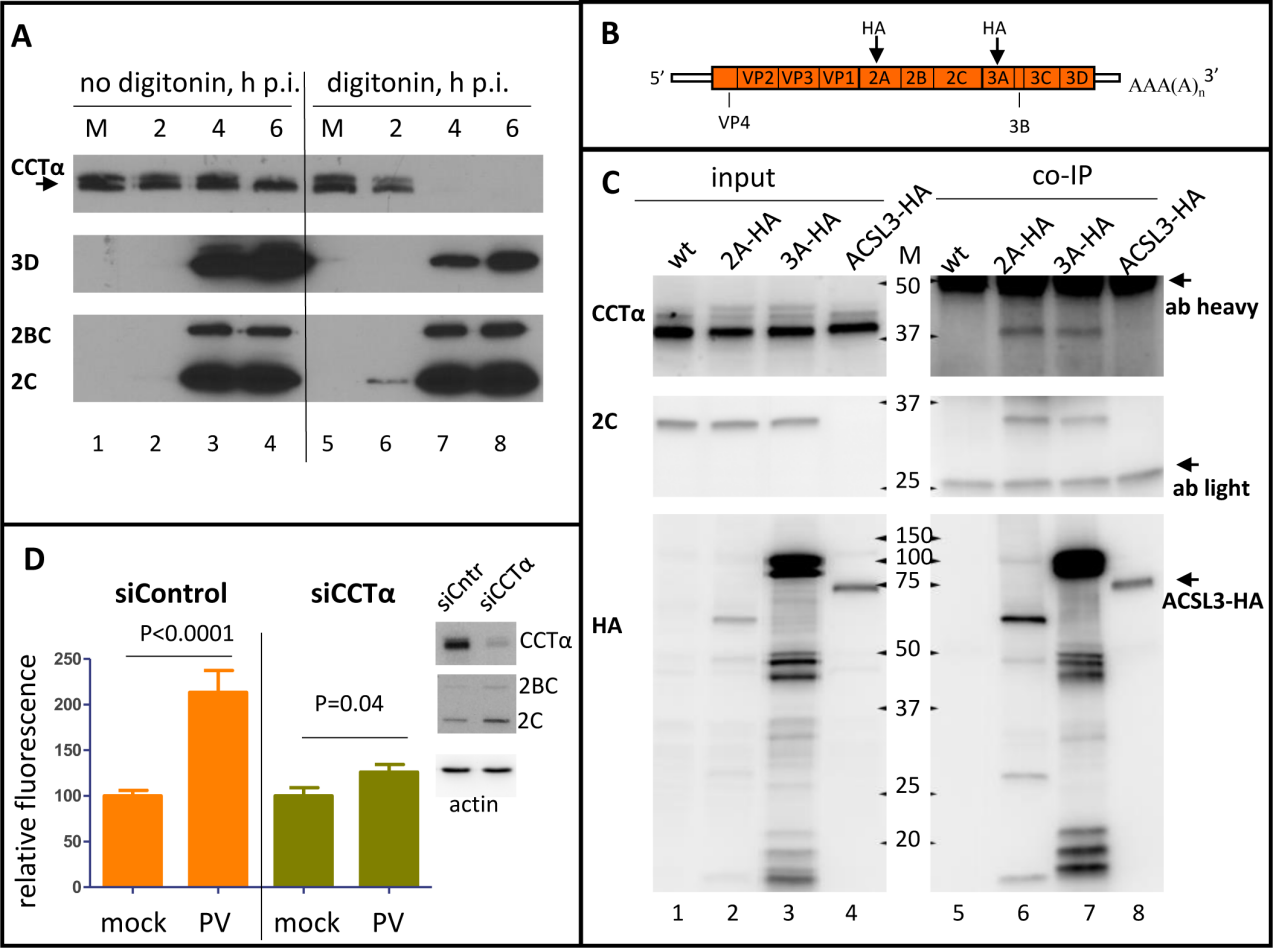
CCTα associates with the replication organelles and is important for controlling the activation of phospholipid synthesis upon infection. **A.** Western blot showing changes in phosphorylation status and localization of CCTα upon infection. HeLa cells infected with 10 PFU/cell of poliovirus were treated with digitonin and collected for Western blot at the indicated times p.i. (lanes 6–8); control cells (lanes 2–4) underwent the same treatment but without the detergent. Arrow indicates dephosphorylated activated form of CCTα. Mock-infected cells (lanes 1 and 5) were collected at 6 h. Proteins were detected on the same membrane after stripping previous antibodies. **B.** Scheme of poliovirus genome with the sites of HA antigen insertions in the 2A or 3A sequence indicated. **C.** Top panel: CCTα signal from co-IP with anti-HA antibodies from lysates of HeLa cells infected at an MOI of 10 PFU/cell with either wt poliovirus (lanes 1 and 5), 2A-HA (lanes 2 and 6), 3A-HA (lane 3 and 7), or cells transfected with a plasmid expressing ACSL3-HA (lanes 4 and 8). Infected cells were processed at 6 h p.i. Bottom panel shows western blot of the same membrane probed with anti-HA antibodies. Multiple products of the viral polyprotein processing containing HA antigens (lanes 2, 3, and 6, 7) as well as a single band of ACSL3-HA (lanes 4 and 8) are detected. Positions of heavy and light antibody chains in co-IP samples are indicated. Input material on the western constitutes 5% of the lysate taken for co-IP. **D.** Cells with siRNA-knockdown expression of CCTα were infected with poliovirus at 10 PFU/cell and at 4 h post-infection, they were provided with a fluorescent long chain FA analog. The accumulation of fluorescence reflects the activation of lipid synthesis. The data are normalized to incorporation of the fluorescent long chain FA in corresponding mock-infected controls.

To analyze the dynamics of CCTα translocation during the time course of infection, we performed western blot analysis of lysates from HeLa cells infected with an MOI of 50 PFU/cell of poliovirus (so that all the cells are infected simultaneously), treated or non-treated with digitonin before the lysis. Treatment with digitonin removes cholesterol from the membranes, thus making the cholesterol-rich plasma membrane permeable while leaving cholesterol-poor membranes of intracellular organelles, including the nuclear envelope, relatively intact. Western blot revealed CCTα signal in two bands (Fig 3A), apparently corresponding to the phosphorylated and dephosphorylated forms (Fig 3A, arrow) of the protein observed previously [39, 40]. It is believed that the dephosphorylated protein represents a more active form of the enzyme with a higher membrane affinity and increased sensitivity to the activation signals [41]. As infection progressed, there was a redistribution between the two forms of CCTα with the higher-running phosphorylated form disappearing, while the lower- running, dephosphorylated form was accumulating (Fig 3A, compare lane 1, mock, with lanes 2–4, infected). Digitonin treatment did not significantly affect the total recovery of CCTα from the mock-infected cells, confirming that most of the protein is confined within the nuclei (Fig 3A, compare lanes 1 and 5). In the lysates from digitonin-treated infected cells, however, even at 2 h p.i. there was a significant reduction in the amount of recovered CCTα which was not detected at all in digitonin-treated infected cells at later time points (Fig 3A, lines 6, 7, and 8). Staining of the membrane for a soluble viral protein 3D, an RNA-dependent RNA polymerase, demonstrated that it was significantly lost upon digitonin treatment; some amount of 3D recovered from digitonin-treated cells is likely incorporated in membrane-associated replication complexes (Fig 3A and 3D panel, compare lanes 3,4 and 7,8). At the same time, the membrane-associated viral proteins 2C and 2BC were recovered similarly from both digitonin-treated and non-treated cells, confirming that treatment conditions preserved intracellular membranes (Figs 3A and 2C panel, compare lanes 3, 4 and 7, 8).

The immunofluorescence data (Fig 2A) suggested that CCTα was interacting with some cytoplasmic structures in infected cells, however since the protein was lost after digitonin treatment, this interaction was not very strong. We hypothesize that this behavior may reflect activation of CCTα known to be accompanied by its translocation from the nucleus and transient association with membranes [42–44].

To see if CCTα is recruited to the replication complexes we performed a co-immunoprecipitation assay. HeLa cells were infected with polioviruses that had an HA tag either in 2A or in 3A protein with an MOI of 10 PFU/cell (Fig 3B). Because poliovirus RNA is translated into one polyprotein which undergoes proteolytic processing, HA tags will be found not only in 2A or 3A, but also in multiple intermediate cleavage products (Fig 3C, HA panel). As controls we used either cells infected with wild type (wt) poliovirus, or cells transfected with a plasmid expressing an HA-tagged long chain acyl-CoA synthetase 3 (ACSL3-HA), an enzyme responsible for activation of long chain FAs and involved in the lipid metabolism [45]. Both HA-tagged viruses replicated to the same level as the wt control as evidenced by similar expression of the viral 2C protein in all samples (Figs 3C and 2C panel, input). The cells were lysed at 6 h p.i. and processed for immunoprecipitation with anti-HA antibodies, and the recovered material was analyzed with anti-CCTα antibodies. CCTα was recovered only in co-IP samples from cells infected with 2A- or 3A-tagged polioviruses but not from cells infected with wt poliovirus or expressing HA-tagged ACSL3 (Fig 3C, CCTα panel). We also detected a viral protein 2C, a known component of the poliovirus replication complex, in both co-IP samples from cells infected with HA-tagged viruses, thus confirming the specificity of co-IP conditions and indicating that CCTα is a part of multi-subunit replication complexes including viral and cellular proteins (Figs 3C and 2C panel, co-IP). To see if CCTα is directly responsible for activation of membrane synthesis upon infection, we knocked down its expression using siRNA. siRNA-treated cells were infected with poliovirus at an MOI of 10 PFU/cell and at 4 h p.i. the cells were provided with a fluorescently-labeled long chain FA analog Bodipy C4/C9. Quantitation of the fluorescent signal of the FA incorporated into cellular lipids demonstrated significant activation of lipid synthesis in infected cells treated with control siRNA, similar to that described previously [26]. In the cells treated with CCTα-specific siRNA, the increase in lipid synthesis almost disappeared (Fig 3D), while the viral replication was not affected as evidenced by the similar accumulation of the viral proteins in both samples (Fig 3D, western blots). The detailed investigation of the role of phospholipid synthesis in poliovirus infection is described further in the paper. It should be noted that due to its predominantly nuclear localization, CCTα has a long turnover period, and the siRNA-knockdown was not complete, which likely explains the residual activation of the lipid synthesis upon infection (Fig 3D, western blots). Collectively, these data suggest that poliovirus infection induces strong post-translational activation of the cellular pool of CCTα, accompanied by its translocation from the nuclei and association with the replication membranes, which is likely necessary to sustain massive upregulation of phospholipid synthesis.

### Lipid droplets provide the bulk of long chain FA supply to support infection-specific PC synthesis

**Fig 4:**
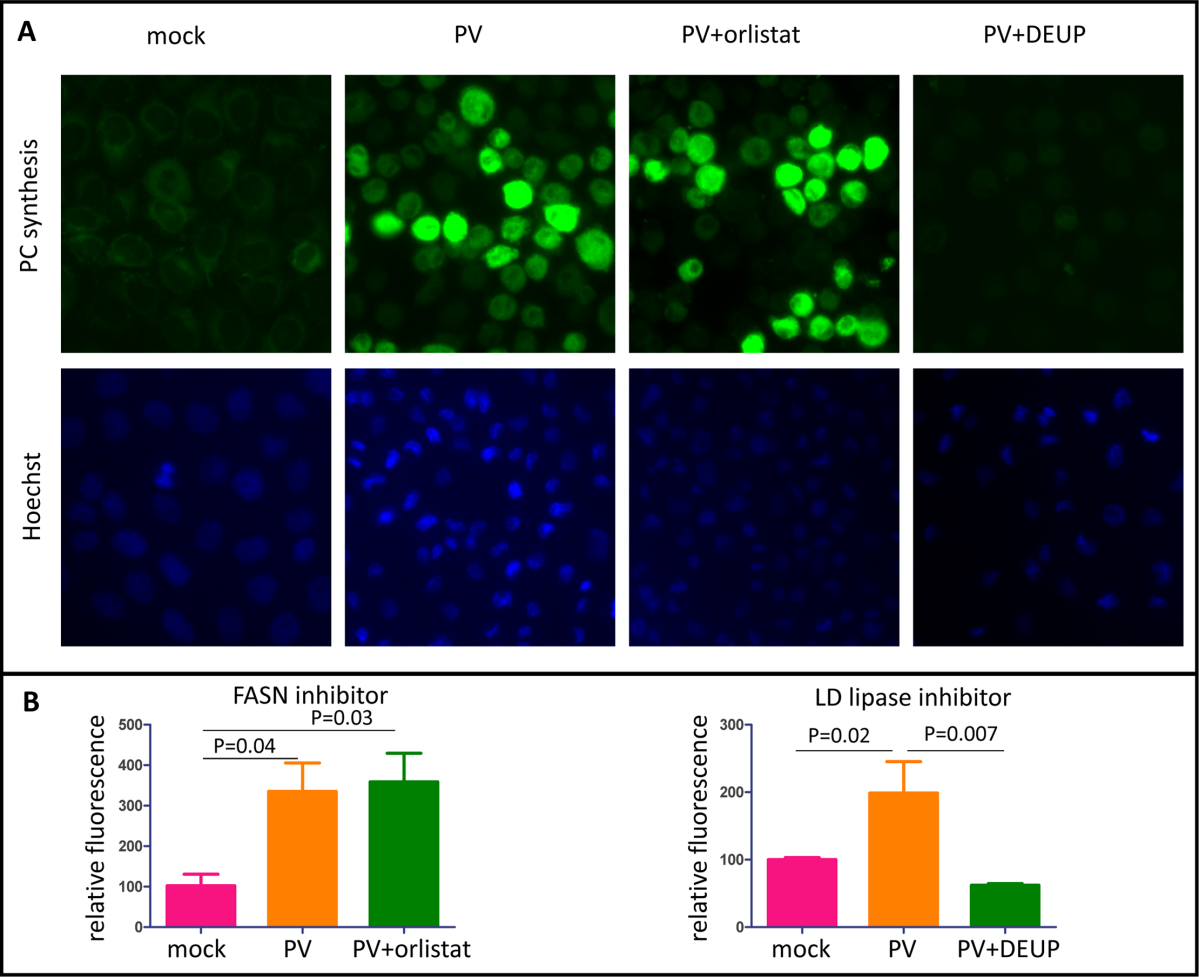
FAs from lipid droplets sustain activated phospholipid synthesis in infected cells. **A.** Incorporation of a choline analog propargylcholine into the membranes of infected cells. HeLa cells were infected with poliovirus at an MOI of 10 PFU/cell, and were incubated with the indicated inhibitors after infection in balanced Earle solution. Orlistat is an inhibitor of *de novo* FA synthesis by FASN, DEUP is an inhibitor of mobilization of neutral lipids stored in lipid droplets. At 5 h p.i., the incubation medium was replaced with fresh pre-warmed balanced Earle solution containing propargylcholine. The cells were fixed at 6 h p.i. and processed for click-chemistry-based detection of incorporated propargylcholine. **B.** Quantitation of propargylcholine incorporation by HeLa cells infected and processed as in A.

Activation of phospholipid synthesis requires an increased supply of long chain FAs. The mammalian cells can obtain them from three major sources: import from the incubation medium (serum) [46, 47]; *de novo* synthesis via fatty acid synthase (FASN)-dependent pathway [48, 49]; or hydrolysis of triglycerides and cholesterol esters stored in lipid droplets [50]. We previously demonstrated that activation of phospholipid synthesis does not require presence of serum in the incubation medium [27], indicating that membrane synthesis upon infection could be sustained by internal cellular resources. To evaluate the relative contribution of FAs from the FASN-dependent *de novo* synthesis and those released from lipid droplets, we blocked them individually and assessed the activation of PC synthesis upon infection. The activity of FASN was blocked by orlistat, a compound that inhibits the thioesterase domain of the multifunctional enzyme [51]. Activity of all lipid droplets-associated lipases can be efficiently blocked by diethylumbelliferyl phosphate (DEUP) [52–56]. In some cell types, microautophagy (lipophagy) can also significantly contribute to turnover of neutral lipids stored in lipid droplets [52]. This activity is sensitive to inhibitors of lysosome acidification such as bafilomycin [57]. To quantitate the activation of PC synthesis, we adopted a method of propargylcholine labeling of phospholipids described in [58]. Cells metabolize this compound similarly to choline and incorporate it into newly-synthesized phospholipids. Subsequent click-chemistry reaction between the alkyne group of propargylcholine with an azide derivative of a fluorescent dye allows quantitative measurement of phospholipid synthesis. First, we evaluated the effect of the inhibitors of FA fluxes on a single round of poliovirus replication upon infection of HeLa cells with an MOI of 50 PFU/cell. In these conditions, none of the treatments (incubation of cells with DEUP, orlistat, or bafilomycin) had significant effect on the viral propagation (S1A Fig), thus permitting a direct comparison of the contribution of each source of long chain FAs in infection-specific upregulation of phospholipid synthesis. In infected cells incubated in the presence of orlistat, we observed the same level of upregulation of propargylcholine incorporation as in control cells, thus ruling out the significant contribution of newly synthesized FAs into the overall balance of phospholipid synthesis (Fig 4A and 4B). On the other hand, incubation of infected cells in the presence of DEUP severely inhibited activation of phospholipid synthesis, indicating that lipid droplets provide the main source of material to support membrane biogenesis in infected cells (Fig 4A and 4B). Since bafilomycin did not have an inhibitory effect on activation of membrane synthesis (S1B Fig), we conclude that activity of lipid droplets-associated lipases, but not lipophagy is responsible for the release of FAs from lipid droplets in infected cells.

### Poliovirus infection induces recruitment of lipases to lipid droplets and activates lipolysis of neutral lipids

**Fig 5:**
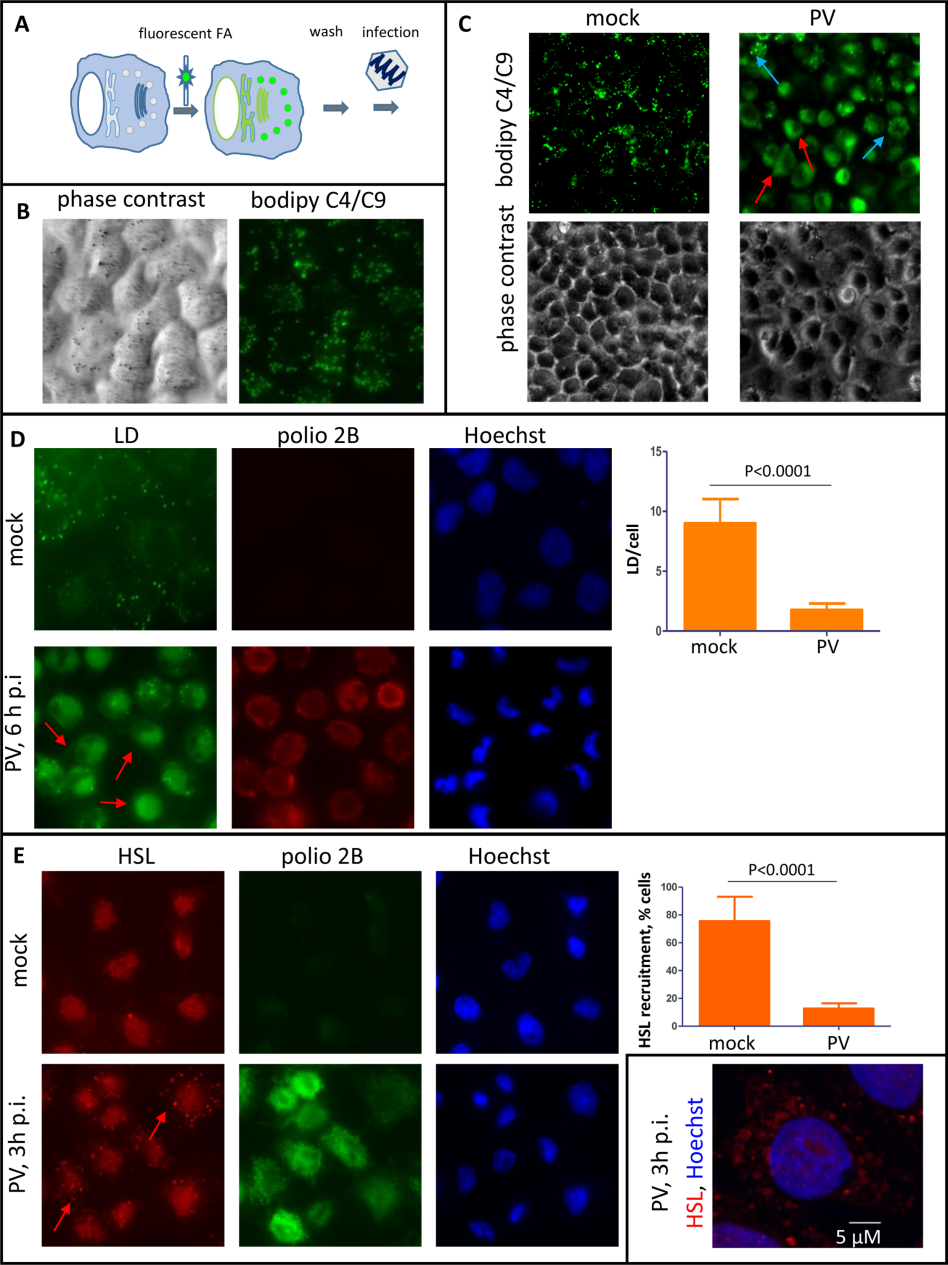
Poliovirus infection stimulates hydrolysis of neutral lipids in lipid droplets. **A.** Scheme of the experiments shown on panels B and C. HeLa cells were pre-incubated with a fluorescent FA analog before infection so that the molecule is incorporated into the neutral lipids stored in lipid droplets. **B.** Incorporation of a fluorescent long chain FA into lipid droplets of non-infected cells upon 1 h of incubation. **C.** HeLa cells pre-treated with Bodipy 500/510 C4/C9 for 1h were infected (mock-infected) with poliovirus at an MOI of 10 PFU/cell and incubated without the fluorescent FA for 6 h p.i. Red arrows indicate cells with redistribution of the fluorescent FA into the membranes of replication organelles, blue arrows indicate cells with residual fluorescence in lipid droplets. **D.** Disappearance of lipid droplets by the end of the poliovirus replication cycle. HeLa cells were infected (mock-infected) with poliovirus at an MOI of 10 PFU/cell and at 6 h p.i. they were fixed and processed for immunofluorescent analysis of a viral antigen 2B and stained with a lipid droplets-specific dye Bodipy 493/503. Arrows indicate infected cells where lipid droplets are no longer detectable. **E.** Recruitment of HSL to lipid droplets early during the poliovirus replication cycle. HeLa cells were infected (mock-infected) with poliovirus at an MOI of 10 PFU/cell and at 3 h p.i. they were fixed and processed for immunofluorescent analysis of a viral antigen 2B and HSL. Arrows indicate recruitment of HSL to lipid droplets. Inset shows a high magnification confocal image of HSL recruitment to lipid droplets in poliovirus-infected cells.

To directly observe if long chain FAs from the neutral lipids stored in lipid droplets are used in the development of the poliovirus membranous replication organelles, we incubated HeLa cells for 1 h before infection in the presence of a fluorescent long chain FA analog Bodipy C4/C9. After this incubation, the medium containing the fluorescent FA was removed, and the cells were extensively washed before infection to permit observation of redistribution of already incorporated fluorescent FA upon infection (Fig 5A). As expected, in uninfected cells this molecule is preferentially incorporated into neutral lipids and is targeted to lipid droplets (Fig 5B). In mock-infected cells, the fluorescence was still mostly exclusively confined to lipid droplets by 6 h of incubation (Fig 5C). In cells infected with poliovirus at an MOI of 10 PFU/cell, we observed a massive translocation of fluorescence from lipid droplets into the perinuclear region, characteristic of localization of poliovirus replication complexes (Fig 5C, red arrows), confirming that long chain FAs released from lipid droplets indeed support the development of the replication organelles. Note that some infected cells still show fluorescent signal residing in lipid droplets (Fig 5C, blue arrows). Thus, our data show that neutral lipids stored in lipid droplets are mobilized upon poliovirus infection and sustain activation of membrane synthesis.

To monitor the utilization of endogenous lipids in lipid droplets, we infected HeLa cells with poliovirus at an MOI of 10 PFU/cell and stained them with Bodipy 493/503, a lipid droplet-specific dye, at 6 h p.i. In mock-infected cells multiple lipid droplets were detected in virtually every cell as bright green dots, while in infected cells the amount of lipid droplets was significantly lower, and in many cells the typical dot-like staining of lipid droplets was not detected at all (Fig 5D, red arrows). Rather, the Bodipy 493/503 signal was increased in the perinuclear area, likely reflecting massive development of the replication membranes. Quantitative analysis confirmed a significant reduction of lipid droplets per cell from about nine in mock-infected control to less than two in infected cells, suggesting that many lipid droplets are completely exhausted upon infection (Fig 5D).

Mobilization of neutral lipids stored in lipid droplets depends on lipid droplet-associated lipases, such as adipocyte triglyceride lipase (ATGL), hormone sensitive lipase (HSL), and monoglyceride lipase (MGL) which release FAs from the glycerol backbone of triglycerides, with HSL being also active on cholesterol esters, reviewed in [59]. We monitored recruitment of these lipases to lipid droplets upon infection. We did not see any significant association of MGL signal with lipid droplets in either infected or control cells (S2A Fig). The HSL signal in mock-infected HeLa cells was mostly localized in the nuclear area, with about 15% of cells having lipid droplets-associated signal (Fig 5E). A significant nuclear signal of HSL in HeLa cells is consistent with the previously reported data of a nucleus-associated pool of HSL in the epithelial cells of mammalian female reproductive tract [60] as well as with the antibody manufacturers data. On the other hand, almost 80% of cells infected with 10 PFU/cell of poliovirus at 3 h p.i. demonstrated multiple bright HSL-positive cytoplasmic dots reflecting recruitment of the enzyme to lipid droplets (Fig 5E, red arrows and inset). We also observed a significant recruitment of ATGL to lipid droplets upon infection, although it was less pronounced than HSL and was detected at 3 h p.i. in about 20% of infected cells compared to less than 5% in mock-infected control (S2B Fig). Thus, poliovirus infection induces strong recruitment of major lipases controlling utilization of neutral lipids to lipid droplets, which is likely responsible for the increasing supply of long chain FAs required to feed the activated phospholipid synthesis in infected cells.

### The first cycle of poliovirus replication is not significantly affected by inhibition of phospholipid synthesis

**Fig 6:**
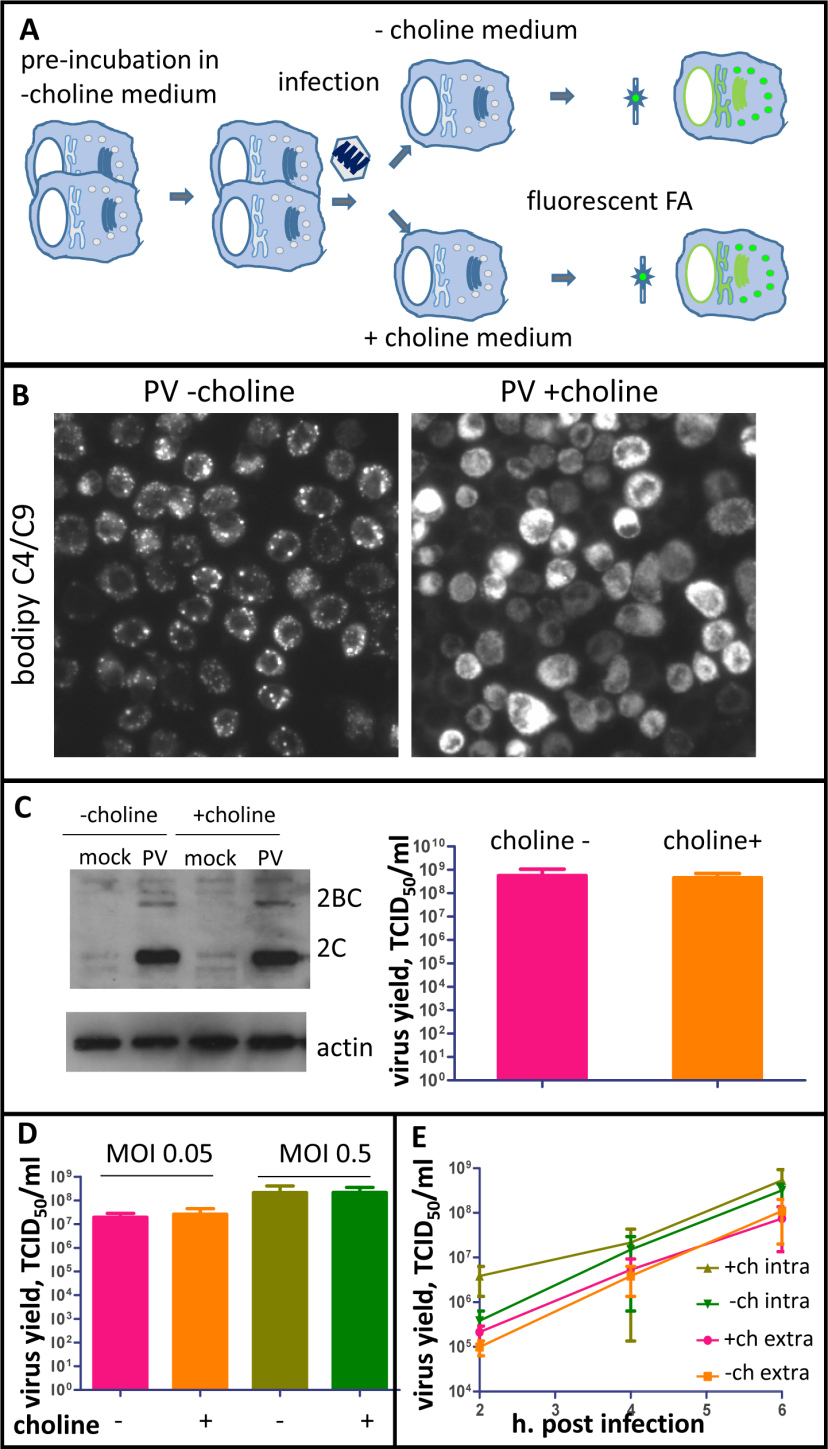
Inhibition of PC synthesis restores neutral lipid synthesis in infected cells but does not significantly affect the first cycle of poliovirus replication. **A.** Scheme of the experiments shown on panels B-D. HeLa cells pre-incubated in choline-free medium for ~72h were infected with poliovirus and were incubated after infection either in choline-free or choline-supplemented medium. **B.** Incorporation of the fluorescent long chain FA analog added to choline-deprived HeLa cells infected with an MOI of 10 PFU/ml of poliovirus at 4 h p.i. for 1 h. In cells incubated in choline-free medium, it redistributes to neutral lipids in lipid droplets (left panel), while in cells incubated with choline-supplemented medium it is used for membrane synthesis (right panel). **C.** Accumulation of the viral proteins 2C and 2BC (left panel) at 4 h p.i. and total virus yield (right panel) at 6 h p.i. did not depend on whether previously choline-deprived cells were incubated in choline-free or choline-supplemented medium after infection. Actin shown as a loading control. **D.** Total virus yield at 6 h p.i. upon infection of choline-deprived cells at low MOIs of 0.05 and 0.5 does not depend on whether the cells were incubated in choline-free or choline-supplemented medium after infection. **E.** Presence of choline in the incubation medium also did not affect the dynamics of accumulation of extracellular (extra) and intracellular (intra) progeny virus upon infection of choline-deprived HeLa cells. Virus yield at the indicated time points was determined after infection of choline-deprived HeLa cells with an MOI of 10 and incubation after infection in choline-free or choline-supplemented media.

To understand the role of activation of membrane biogenesis in poliovirus infection, we started from analyzing the effects of its inhibition on the first cycle of viral replication. To block new membrane synthesis, the cells were pre-incubated before infection in a choline-free medium to exhaust the endogenous choline pool. In conditions of cell culture, synthesis of all major structural phospholipids is regulated by the availability of choline, which cannot be synthesized by cells and has to be provided in the medium. Choline is first incorporated into PC, which in turn can be converted into phosphatidylserine and phosphatidylethanolamine, reviewed in [61, 62]. To validate the choline depletion approach, we monitored the incorporation of a fluorescent long chain FA analog Bodipy C4/C9. HeLa cells seeded at a low density were incubated for ~72 hours in a choline-free medium so that they could divide and grow, thus exhausting the intracellular choline depot for new membrane synthesis. Importantly, choline deprivation did not affect the overall cell viability for up to 4 days (S3 Fig), in accordance with previous observations showing that cells can tolerate choline deprivation for prolonged period of time [63, 64]. On the third day, choline-deprived cells were infected with poliovirus at an MOI of 10 PFU/cell, and were either incubated in a choline-free or a choline-supplemented medium after infection. At 5 h p.i., the media in both samples were supplemented with Bodipy C4/C9 for 1 h (Fig 6A). As can be seen in Fig 6B, incorporation of the fluorescent FA analog into cellular structures of infected cells incubated in choline-free or choline supplemented media was drastically different. In a choline-supplemented medium, the fluorescence was distributed in perinuclear rings, typical of localization of poliovirus membranous replication organelles, indicating activation of new membrane synthesis. In infected cells incubated in choline-free medium, however, the fluorescence was confined to lipid droplets, showing that the flux of long chain FAs was directed almost exclusively toward synthesis of neutral lipids. Thus, choline depletion prevented activation of membrane synthesis, as predicted. This result also confirms that the switch from neutral to phospholipid synthesis upon picornavirus infection should be attributed to activation of CCTα and increasing PC synthesis, and not to inhibition and/or degradation of neutral lipid synthesizing enzymatic machinery.

At the same time, the level of virus replication was unaffected in a choline-free medium, as evidenced from the western blot showing accumulation of the viral proteins 2C and 2BC and the viral titer (Fig 6C). Sometimes we observed a slight suppression of viral protein accumulation in choline-depleted cells, but that did not translate into a statistically significant difference of the virus titer by the end of infection (S1C Fig).

We also analyzed if choline deprivation may affect the first replication cycle if the cells are infected at a low MOI. HeLa cells pre-incubated in a choline-free medium for 72 h were infected with an MOI of 0.5 or 0.05 PFU/cell, and were incubated after infection in choline-free or choline-supplemented media for 6 h. There was no significant difference in the virus propagation in either condition (Fig 6D), confirming that the first cycle of infection proceeds similarly in choline-deprived or choline-supplemented cells.

Next, we compared the dynamics of synthesis of infectious poliovirus in one replication cycle in cells incubated in a choline-depleted or a choline-supplemented medium. We separately analyzed accumulation of progeny virions inside the cells and their release into extracellular medium, as it is likely that inhibition of membrane synthesis could affect the process of non-lytic picornavirus release described earlier [65–67]. HeLa cells pre-incubated in a choline-free medium for ~72 hours were infected with poliovirus at an MOI of 10 PFU/cell, and were incubated after infection in either a choline-free or a choline-supplemented medium. Extracellular and intracellular virus were collected at 2, 4 and 6 h p.i. We did not observe any significant effect of membrane synthesis on either intracellular virus accumulation or on the amount of the virus recovered from the medium (Fig 6E). Thus, activation of membrane synthesis is not essential for the first round of poliovirus replication.

### Phospholipid synthesis sustains structural development of the poliovirus replication organelles

**Fig 7:**
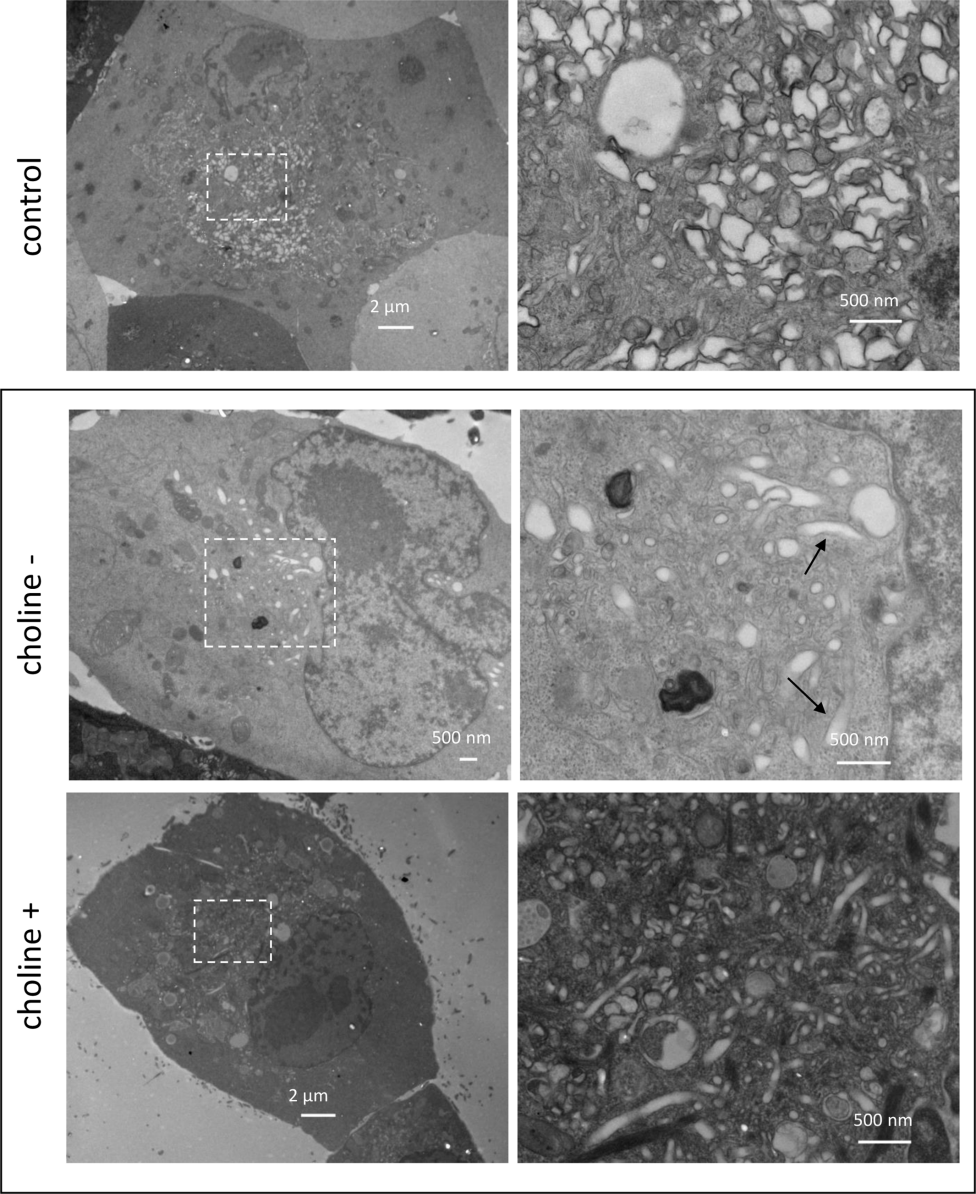
Inhibition of phospholipid synthesis impedes structural development of replication organelles. HeLa cells were infected with 10 PFU/cell of poliovirus and processed for transmission EM imaging at 4 h p.i. The cells were incubated in either normal serum-supplemented medium before and after infection (control), or were pre-incubated for ~72h in choline-free medium, and after infection were incubated in choline-free (choline-), or choline-supplemented (choline +) medium. Areas outlined on low magnification images on the left are shown at high magnification on the right. Arrows on high magnification choline- panel indicate enlarged ER-like structures.

The ability of poliovirus to replicate equally well in conditions of activated and inhibited phospholipid synthesis allowed for direct investigation on whether the new membrane synthesis or remodeling of pre-existing cellular membranes supports structural development of poliovirus replication organelles. HeLa cells were pre-incubated for ~72 h in a choline-free medium, infected with 10 PFU/cell with poliovirus, and were incubated after infection in either a choline-free or a choline-supplemented medium until 4 h p.i. For comparison, a control sample was prepared with cells that did not undergo choline deprivation treatment and were maintained in a serum-supplemented complete medium. In these conditions (choline-free, choline-supplemented, and complete medium with serum), accumulation of the viral proteins was similar (S1C Fig), confirming that inhibition of PC synthesis did not negatively affect viral replication. Infected cells incubated in a complete serum-containing medium demonstrated typical development of the poliovirus replication organelles with large clusters of tightly associated heterogeneous membranous compartments appearing in the perinuclear region of the cells (Fig 7, control). The cells infected upon choline depletion and incubated in a choline-free medium post infection revealed detached vesicles or tubules sparsely distributed in the cellular cytoplasm, often found close to the ER tubules (Fig 7, choline-). Elongated membranous compartments, likely to be dilated ER tubules were also present (Fig 7, arrows). Addition of choline largely restored the complexity of the membranous replication structures, although their appearance was somewhat different compared to the cells that did not undergo choline deprivation treatment. Membranous compartments in choline-supplemented cells were smaller and more loosely associated, and contained more elongated tubular-like structures (Fig 7, choline+). Thus, activation of phospholipid synthesis, not the remodeling of the pre-existing membranes, is responsible for the massive growth of the replication organelles.

### Extensive membrane remodeling protects viral replication complexes

**Fig 8:**
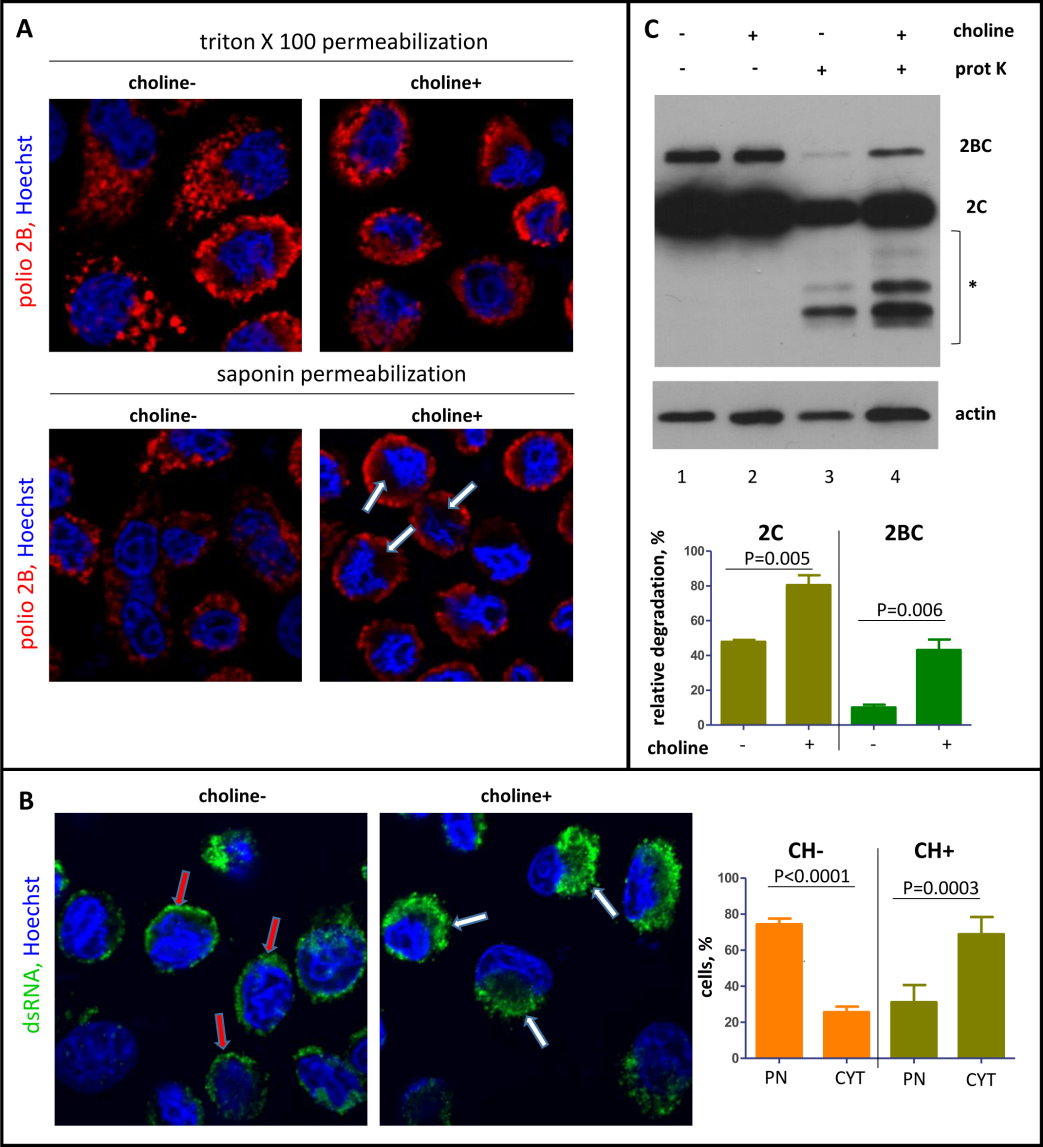
Inhibition of phospholipid synthesis affects the accessibility of the replication complexes. HeLa cells after choline deprivation were infected with poliovirus at an MOI of 10 PFU/cell and incubated in a choline-free or a choline-supplemented medium after infection. **A.** Confocal images of the cells permeabilized with either 0.2% Triton X100 (strong permeabilization) or 0.02% saponin (mild permeabilization) and stained for a viral antigen 2B (red). Nuclear DNA is stained with Hoechst 33342 (blue). Arrows indicate areas protected upon mild permeabilization in cells incubated with choline-supplemented medium. **B.** Visualization of dsRNA in HeLa cells processed and infected as in A after Triton X100 permeabilization. Red arrows indicate dsRNA association with the nuclear envelope (PN) in choline-deprived cells; white arrows show big perinuclear blobs (CYT) of dsRNA signal reflecting normal development of replication membranes in choline-supplemented cells. **C.** HeLa cells were infected with poliovirus at an MOI of 10 PFU/cell after choline deprivation and incubated in a choline-free or a choline-supplemented medium for 4 h p.i. The cells were permeabilized with digitonin and treated (lines 3 and 4) or non-treated (lines 1 and 2) with proteinase K. * indicates protein fragments generated upon proteinase K treatment. Actin is shown as a loading control.

EM images of the replication structures formed in conditions of the inhibited membrane synthesis suggested that the viral replication complexes should be less protected and more accessible from the cytoplasm. To test this assumption, we investigated the accessibility of a viral antigen 2B, present in several proteins in the replication complex, in an immunofluorescence assay. When immunostaining was performed in conditions of thorough permeabilization of membranes with 0.2% Triton X100, infected cells incubated in a choline-supplemented medium demonstrated typical continuous perinuclear distribution of the viral antigen, while in cells incubated without choline, viral-specific signals were scattered in separate foci throughout the cytoplasm, consistent with the EM data (Fig 8A, triton panel). In conditions of mild permeabilization with 0.02% saponin, which leaves the membranes relatively intact, the overall appearance of the viral replication structures in cells incubated in choline-free medium was similar to those permeabilized with Triton X100, indicating that membranes were not concealing the replication sites. On the other hand, in cells incubated in choline-supplemented medium, the antibodies upon mild permeabilization could access only the periphery of the perinuclear cluster of the replication membranes, leaving in many cells a dark protected area around the nucleus (Fig 8A, saponin panel, arrows). To further characterize the effect of inhibition of membrane synthesis on the development of replication organelles, we monitored distribution of dsRNA, an intermediate product in the replication cycle of (+)RNA viruses, as well as redistribution of GBF1 and PI4KIIIβ, two components of the host secretory pathway known to be recruited to poliovirus replication complexes [18, 20]. As expected, both cellular proteins were found to similarly redistribute in cells depleted of or supplemented with choline, since protein-protein interactions are unlikely to be affected by inhibition of membrane synthesis (S3 Fig). The distribution of dsRNA, however was significantly different. In cells incubated in a choline-supplemented medium, the dsRNA signal was found in large perinuclear blobs, reflecting normal development of the replication membranes, while in the absence of choline, dsRNA was mostly concentrated in a tight circle around the nuclear envelope (Fig 8B). These data imply that activation of membrane synthesis is important for proper development of the replication organelles, and that interfering with phospholipid synthesis at any step of the metabolic pathway should have similar phenotype in infected cells. Indeed, when we inhibited the hydrolysis of neutral lipids in lipid droplets with DEUP [52–56], thus blocking the supply of long chain FAs, we observed similar defects in the development of the replication organelles as in cells deprived of choline. In the EM images of infected cells incubated in the presence of DEUP, the replication organelles appeared scattered throughout the cytoplasm and did not fuse into a tight perinuclear cluster. Likewise, in an immunofluorescent assay a viral antigen 2B was distributed in separate dots rather than in continuous perinuclear rings as in control cells (S4 Fig).

To biochemically assess the difference in accessibility of the viral proteins in conditions of activated and inhibited membrane synthesis, HeLa cells were pre-incubated in the absence of choline for ~48 h, infected with poliovirus at an MOI of 10 PFU/cell, and incubated after infection in either a choline-free or a choline-supplemented medium. At 4 h p.i., the cells were treated with digitonin, a mild detergent permeabilizing plasma membrane but leaving the intracellular membranes relatively intact, similar to the experiment previously shown on Fig 3A, and after permeabilization the cells were treated with proteinase K. Fig 8C demonstrates that while the level of the viral proteins 2B and 2C accumulated in infected cells was the same regardless of the presence of choline (Fig 8C, lanes 1 and 2), the proteins in cells incubated in the absence of choline were more accessible to proteinase K treatment (Fig 8C, lanes 3 and 4). Thus, activation of phospholipid synthesis is important for the development of the replication organelles and defines their morphological characteristics, affecting the accessibility of the viral replication complexes.

### Inhibition of phospholipid synthesis blocks poliovirus propagation in multiple cycles of infection and increases the efficiency of cellular anti-viral mechanisms

**Fig 9:**
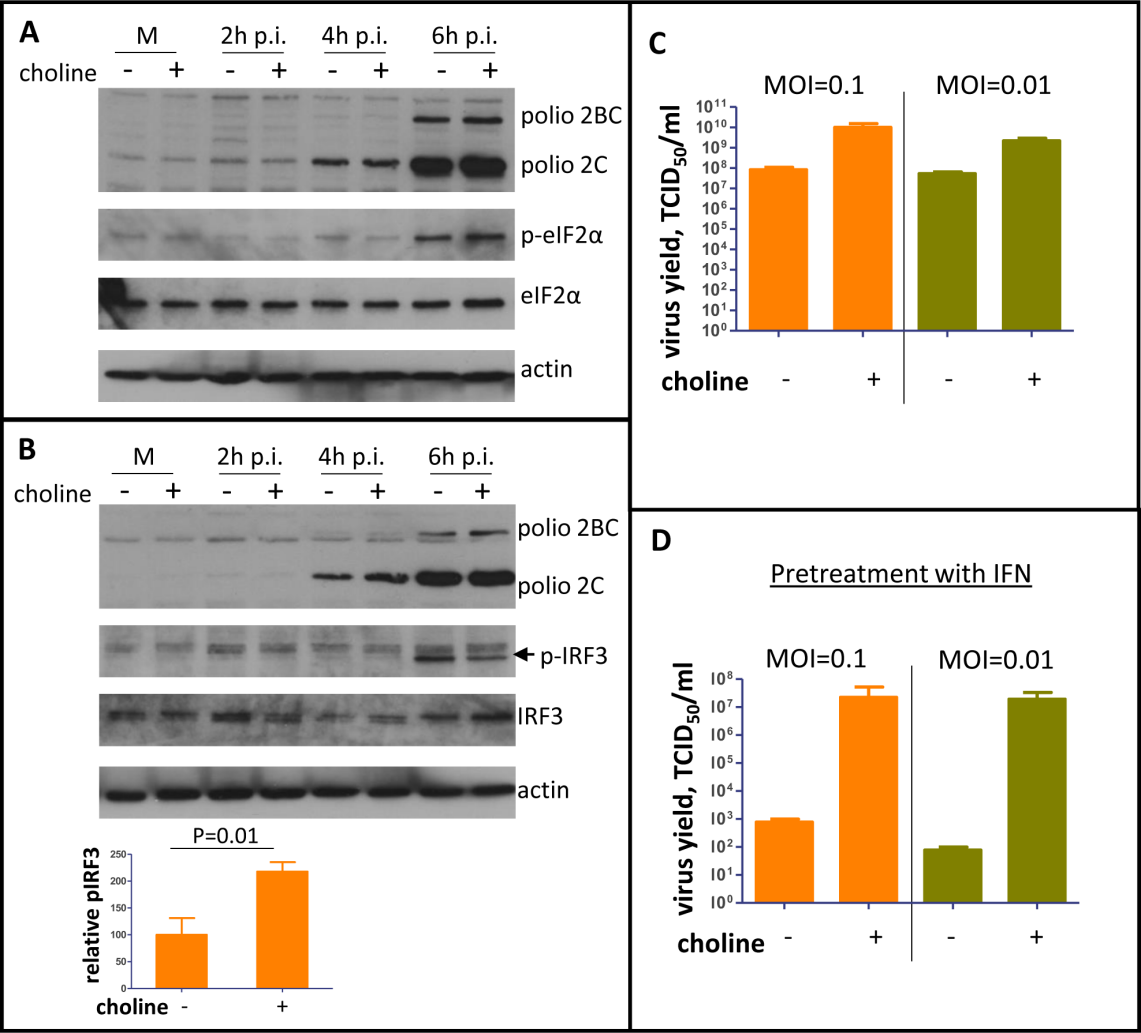
Inhibition of membrane synthesis promotes cellular anti-viral signaling and impedes poliovirus propagation in multiple cycles of infection especially in conditions of pre-activated anti-viral response. **A** and **B.** HeLa cells were pre-incubated in choline-free medium for ~72h, they were infected with poliovirus at an MOI of 10 PFU/cell and incubated in either a choline-free- or a choline-supplemented medium for the indicated time p.i. Phosphorylated IRF3 is indicated by the arrow. Actin is shown as a loading control. **C.** HeLa cells were pre-incubated in choline-free medium for ~72 h, infected with poliovirus at an MOI of 0.1 or 0.01 PFU/cell, and were incubated in either a choline-free or a choline-supplemented medium for another 24 h. Total virus yield is shown. **D.** HeLa cells were pre-incubated in choline-free medium for ~60h followed by ~12 h of incubation with 20u of universal type I interferon in choline-free medium. After that, the cells were infected with poliovirus at an MOI of 0.1 or 0.01 PFU/cell and were incubated in either choline-free or choline-supplemented medium for another 24 h. Total virus yield is shown.

The exposure of the replication complexes to the cytoplasm in conditions of inhibited membrane synthesis suggests that the cellular sensors of infections may also be activated stronger and/or earlier. One of the major triggers of antiviral response to picornavirus infection is dsRNA, an intermediate of viral genome RNA replication [68]. Recognition of dsRNA by several specialized cellular sensors activates a network of protective pathways. Signaling cascades activated by retinoic acid inducible gene (RIG)-like receptors (RLR) RIG-I and MDA5, and toll-like receptors (TLR) 3 and 7, converge on two major branches. One is phosphorylation of transcription factors IRF3/7, which induce expression of interferons α/β. The other is phosphorylation and degradation of Iκβ, resulting in release of transcriptionally active subunits of NF-κβ responsible for activation of expression of pro-inflammatory cytokines, reviewed in [69–71]. Activation of protein kinase R (PKR) by dsRNA induces phosphorylation of a number of substrates including eukaryotic initiation factor 2 α-subunit (eIF2α) leading to inhibition of translation of cellular and viral RNAs [72]. To see if infection-activated membrane synthesis is important for the cellular recognition of infection, we monitored phosphorylation of IRF3, degradation of Iκβ, and phosphorylation of eIF2α. HeLa cells were pre-incubated in a choline-free medium for ~72 hours, infected with poliovirus at an MOI of 10 PFU/cell, and incubated after infection in either a choline-free or a choline-supplemented medium for 6 h. As expected, suppression of membrane synthesis did not affect viral replication in these conditions, as evidenced by similar accumulation of the viral antigen 2C (Fig 9A and 9B). The level of eIF2α phosphorylation was similar in both conditions, suggesting that PKR-dependent pathways are not sensitive to the inhibition of membrane synthesis, at least in these conditions (Fig 9A). Similarly, we did not observe any significant differences in the level of degradation of Iκβ (S5A Fig). At the same time, phosphorylation level of IRF3 detected by western blot was at least two times stronger in infected cells incubated in the absence of choline at 6 h p.i. (Fig 9B), indicating that the membranous scaffold of the replication organelles may be important for suppression of at least some branches of the cellular anti-viral signaling.

IRF3 is a transcription factor controlling the initial steps of the anti-viral response, and even minor changes in its activation may significantly affect expression of multiple genes through subsequent signaling and amplification steps. To compare expression of cellular genes involved in the antiviral response in conditions of permitted and inhibited membrane synthesis, we used a qPCR panel profiling transcripts of 84 genes. In infected cells incubated without choline, we observed a statistically significant increase of transcription of several genes, including IL6 and IL8, as well as components of NFκB and AP-1 transcriptional machinery involved in the inflammatory signaling (S5B and S6 Figs). This suggests that membrane synthesis modulates cellular response to poliovirus infection, which may have important implications in a natural host.

The one-cycle replication experiments, while very informative about the biochemical facets of viral replication, do not fairly reflect the natural infection conditions. In an animal host, infection begins in a few cells from the original virus inoculum, and the virus has to spread to other cells in the body in multiple cycles of replication, virus release, and new infections, accompanied by the mounting of the host anti-viral defenses. To see if activation of membrane synthesis may be important for the virus spread in multiple rounds of infection, we pre-incubated HeLa cells for ~72 h in choline free conditions and infected them with poliovirus at MOIs of 0.1 or 0.01 PFU/cell. After infection, the cells were incubated in a choline-free or a choline-supplemented medium for another 24 h to allow multiple cycles of infections (poliovirus replication cycle in HeLa cells lasts about 6 h). By the end of the experiment, the total virus yield was around two orders of magnitude lower if membrane synthesis was inhibited (Fig 9C). The virus yield in different experiments varied from being ~1.5 to 3 logs lower in choline-deprived than in choline-supplemented cells, likely reflecting the level of the inhibition of phospholipid synthesis. The strong dependence of poliovirus propagation in multiple cycles of infection on activation of membrane synthesis indicates that the cells may mount an efficient anti-viral response and/or that the infection becomes more sensitive to the cellular defense mechanisms if the viral replication complexes are not protected by the membranes.

To test if inhibition of membrane synthesis increases the sensitivity of poliovirus propagation to pre-activated cellular anti-viral program, HeLa cells were pre-incubated for ~60 h in a choline-free medium, and treated overnight with 20 units of universal type I interferon. Such conditions of interferon treatment are very mild and the cells did not show any signs of toxicity. At the same time, expression of interferon-inducible genes was clearly activated (S3B Fig). After the interferon treatment, the cells were infected with poliovirus at MOIs of 0.1 or 0.01 PFU/cell, and were incubated for 24h post infection in either a choline-free or a choline-supplemented medium without interferon. Importantly, in mock-infected cells the presence or absence of choline in the medium did not change expression of interferon-inducible genes (S5C Fig). The infected cells incubated in the presence of choline, in spite of pretreatment with interferon, could support a relatively high level of poliovirus replication (around 1E7 TCID_50_/ml), consistent with a previous report that normally enterovirus replication is not very sensitive to interferon treatment [73]. At the same time, virus propagation was almost completely inhibited in cells incubated in a choline-free medium (1E2-1E3 TCID_50_/ml) (Fig 9D). Thus, activation of membrane synthesis impedes recognition of dsRNA by at least some cellular sensors of infection and permits viral replication in conditions of pre-activated anti-viral response, likely constituting an important component of the virus survival strategy.

## Discussion

The rapid development of the membranous replication structures is one of the longest known but still enigmatic cellular manifestations of picornavirus infection. In cells infected with poliovirus or a related Coxsackie B3 virus, the virus-induced membranes appear as early as 2 h p.i. at the ER-Golgi interface and continue to grow throughout the replication cycle, transitioning from sponge-like membranous clusters to assemblages of double membrane vesicles [11, 74–76]. The unique morphology of the replication organelles implies that mechanism(s) of their formation and/or their composition are different from those supporting membrane architecture in non-infected cells. These novel membranous structures, which may occupy most of the cytoplasmic space by the end of infection, are known to harbor actively replicating viral RNA, and thus are generally referred to as replication organelles. Later in infection, progeny virions are found both inside and outside the membranous vesicles, and the data suggest that the double membrane vesicles accumulated by the end of infection may facilitate virion maturation and spread [22, 65, 77, 78]. Still, our understanding of the mechanistic contribution of the membranous matrix in the viral life cycle is mostly speculative. Development of membranous replication complexes is the hallmark of infection of all (+)RNA viruses of eukaryotes, suggesting that membrane association of the RNA replication and/or virion assembly machinery provides specific benefits in the cytoplasmic environment. On the other hand, it cannot be excluded that massive production of membranes is related to the cellular antiviral response aimed at blocking accessibility of the cellular translational apparatus or other metabolic resources necessary for virus propagation.

Our goals in this study were to understand the mechanism(s) underlying the rapid development of the replication organelles, as well as to identify steps in the viral life cycle sensitive to the inhibition of their growth.

Membranes in mammalian cells consist of three major structural phospholipids: PC accounts for more than 50% of total phospholipid content, phosphatidylethanolamine (PE) for 20–45%, and phosphatidylserine (PS) for 3–15%, depending on cell type [79]. Cells obtain long chain FAs necessary for synthesis of the hydrophobic part of these molecules from three major sources–import of lipids from the extracellular medium (serum), FASN-dependent *de novo* synthesis, or hydrolysis of neutral lipids stored in lipid droplets. We demonstrated here that poliovirus-infected cells, at least in cell culture conditions, rely almost exclusively on lipid droplets for supply of long chain FAs for upregulation of membrane synthesis. Lipid droplets are dynamic cellular organelles that store neutral lipids, mainly triglycerides and cholesterol esters. The neutral lipid core is wrapped in a phospholipid monolayer, and diverse proteins associate with lipid droplets permanently or transiently, balancing the synthesis and hydrolysis of lipids, depending on cellular metabolic demands, reviewed in [50].

Lipid droplets previously have been shown to serve as platforms for structural protein processing and virion assembly of hepatitis C virus and some other flaviviruses, a group of (+)RNA viruses, but it is not known if they contribute to the infection-specific changes of lipid metabolism in flavivirus-infected cells [80]. Interestingly, hydrolysis of cholesterol esters stored in lipid droplets was shown to be important for cholesterol enrichment of the replication organelles of human rhinovirus A16, but not other related picornaviruses [81, 82]. Thus, diverse (+)RNA viruses seem to rely on these cellular organelles at some steps of their life cycles, but whether they share a requirement for lipid droplet-derived long chain FAs for the structural development of the replication organelles remains to be established. We observed recruitment of two major lipases, HSL and ATGL, to lipid droplets in infected cells, which likely explains strong activation of lipolysis and exhaustion of lipid droplets upon infection. Further research is required to understand whether viral proteins are engaged in direct interactions with the lipases or if they activate cellular signaling cascades leading to their recruitment and activation.

Our data contradict with previous reports that FASN activity may be important for picornavirus infection [83, 84]. The discrepancy is likely due to the nature of FASN inhibitors used in previous studies, cerulenin and C75. Both molecules target the ketoacyl synthase domain of FASN resulting in accumulation of cytotoxic malonyl-CoAs, which can inhibit viral replication non-specifically [85–87]. In our study we used orlistat, an anti-obesity drug that inhibits pancreatic lipases, and was later discovered also to irreversibly inhibit the thioesterase domain of FASN, which does not result in accumulation of cytotoxic intermediates of FA synthesis [51]. The conclusions about the role of FASN in different viral systems, based only on the effect of cerulenin and similar compounds should be taken with caution.

How can poliovirus efficiently manipulate cellular lipid synthesizing machinery with very limited genetic resources? Poliovirus infection inactivates transcription and translation of cellular genes [88], thus the metabolic changes in infected cells must rely on post-translational regulation of the enzymes present before infection. In most mammalian cell types, the bulk of PC, the major structural phospholipid, is synthesized through the Kennedy pathway with transfer of the phosphocholine group from CDP-choline to diacylglycerol. On the other hand, diacylglycerol can be converted to a triglyceride molecule upon attachment of the third long chain FA moiety. Such utilization of diacylglycerol in either phospholipid or triglyceride synthesis allows balancing the lipid homeostasis and retargeting the flux of long chain FAs towards membrane synthesis or storage, reviewed in [89]. It has been suggested that production of CDP-choline is the rate-limiting step for activation of PC synthesis upon poliovirus infection [29, 90]. CDP choline is generated by CCTα, which in non-infected cells is largely localized inside the nuclei, and such confinement is important for controlling its activity. The active form of the enzyme is found in the cytoplasm where its activity is fine-tuned by phosphorylation status and binding to certain lipids, reviewed in [44, 91]. In infected cells, we observed a rapid translocation of CCTα from the nuclei to the cytoplasm, accompanied by dephosphorylation of the enzyme, which is consistent with strong activation of CCTα. The massive translocation of CCTα to the cytoplasm depends on the proteolytic activity of the viral protease 2A, a key enzyme responsible for degradation of the nucleo-cytoplasmic barrier in poliovirus-infected cells [38, 92]. The co-IP results suggest that at least a portion of CCTα is associated with the replication membranes and interacts with the viral proteins, which may also directly control its activity.

The massive release of CCTα from the nuclear depot in infected cells would redirect diacylglycerol to PC synthesis and therefore drive the increasing rate of mobilization of neutral lipids in lipid droplets to replenish the exhausted diacylglycerol pool. Supporting this model are our observations that in non-infected cells, overexpression of CCTα is sufficient to redirect the flux of long chain FAs from neutral lipids to phospholipid synthesis, and that depletion of choline, which blocks the CCTα-dependent PC synthesis, leads to restoration of neutral lipid synthesis in infected cells. Given the activating effect of free long chain FAs and diacylglycerol on CCTα activity [42, 93, 94], hydrolysis of lipids stored in lipid droplets and CCTα-driven synthesis of PC would engage in a self-amplifying loop, driving ever-increasing production of PC and massive extrusion of new membranes of the replication organelles.

The translocation of CCTα into the cytoplasm and its association with the membranes may not only be responsible for activation of membrane synthesis but could also contribute to the development of enigmatic convoluted tubular morphology of the replication organelles. Binding of CCTα has been shown to remodel membranous surfaces into elongated tubules of diverse diameters in an *in vitro* system, which was attributed to the amphipathic helix present in the C-terminal part of the enzyme [95].

Activation of phospholipid synthesis is observed in cells infected with diverse (+)RNA viruses, including picornaviruses [23, 25, 96], suggesting that it is an important component of infection. Nevertheless, propagation of poliovirus in one cycle replication experiments in choline-deprived cells was almost indistinguishable from that in control conditions. At the same time, inhibition of phospholipid synthesis had a dramatic effect on the structural development of the replication organelles. Instead of typical clusters of convoluted membranes, the mid-cycle replication organelles in cells that could not synthesize phospholipids consisted of scattered vesicles and elongated ER tubules, resembling structures normally observed very early in infection [74, 97]. These data are strikingly similar to those described in a recently published report that demonstrated that inhibition of a lipid trafficking pathway blocks replication organelle development but does not significantly affect one cycle replication of Coxsackie B3 virus, another enterovirus [98].

Resilience of replication of diverse (+)RNA viruses to the changes of morphology and/or composition of the replication membranes has been documented in many systems. Replication complexes of flock house virus, an insect virus, could be retargeted from mitochondria to the ER [99], and replication complexes of brome mosaic virus, a plant pathogen, could efficiently function either in the context of membrane invaginations or flat membranous sheets [100]. However, such plasticity of replication was registered in highly artificial settings, such as model replication in yeast, or in *in vitro* systems. One should keep in mind that animal viruses, including picornaviruses, have to survive and spread among hosts with fully functional innate and adaptive immunity. Thus, it is likely that many of the aspects of virus-cell interactions have evolved to ensure successful propagation and spread of the virus in natural conditions, rather than to merely support biochemical reactions of the replication of viral genome. Indeed, the defects of the development of the replication organelles in the absence of phospholipid synthesis, well tolerated in one cycle of replication, led to the collapse of viral propagation in multiple cycles of replication.

Clearance of infection by an animal host ultimately depends on the ability of infected cells to detect and communicate their status by expression of an array of signaling molecules. The window when cells could efficiently mount antiviral response to picornavirus infection could be rather short, because these viruses rapidly inactivate cellular transcription and translation [88]. Thus, prevention of infection-induced phospholipid synthesis likely not only extends this period, but also makes the infected cells more sensitive to the effectors of antiviral response. Accordingly, the experiments with mild permeabilization of membranes demonstrated increased accessibility of the viral replication complexes, in the absence of a protective membranous matrix. It has been reported recently that membranous replication organelles of hepatitis C virus hide the replication complexes from the cellular sensors of infection [101]. Thus, the protective function of the membranous replication structures emerges as a strategy shared by diverse (+)RNA viruses. The inevitable reliance of diverse viruses on the same elements of cellular phospholipid synthesizing machinery to support infection-specific membrane synthesis offers multiple targets that can be exploited for broad anti-picornavirus therapeutics.

## Materials and methods

### Cells

Human cervical carcinoma HeLa cell line was obtained from Dr. Ehrenfeld, NIH. The cells were maintained in DMEM, high glucose modification, supplemented with 10% heat-inactivated fetal bovine serum. For choline deprivation studies, the cells were seeded overnight in a 12-well plate in serum-supplemented DMEM at 140000cells/well; the next day they were washed with balanced Earle solution and incubated for ~48 or 72 hours in balanced Earle solution supplemented with MEM amino acid mix and L-glutamine. Upon infection, choline-deprived cells were incubated in the same solution, supplemented in corresponding samples with 25 μM choline chloride.

### Viruses

Poliovirus type I Mahoney strain was propagated in HeLa cells. For experiments performed with choline-deprived cells, virus for inoculum was purified by CsCl gradient, essentially as described in [102], and resuspended in TE buffer (10mM Tris-HCl, pH 8.0 containing 1mM EDTA). Infectious virus was quantified by either plaque assay on HeLa cell monolayer covered by an agarose-solidified medium and expressed in this case like PFU/ml, or by infection of HeLa cells grown in 96 well plates; in this case, the titer is expressed as concentration of inoculum inducing CPE in half of the wells (TCID_50_/ml), calculated by Karber’s formula [103]. For infection, the cell monolayer was washed once with balanced Earle solution, and the virus diluted to the desired MOI in balanced Earle solution buffered with 50μM HEPES, pH 7.3, was incubated with cells at room temperature for 30 min on a rocking platform. After adsorption, the cells were supplemented with the desired medium and incubated at 37C for the indicated time p.i. For collection of extracellular virus, the incubation medium was collected prior to freezing of the cells. For total virus collection, cells were frozen with the incubation medium. The virus was released from cells by three freeze-thaw cycles. Poliovirus with HA antigen insertions into 2A or 3A sequences were described in [27] and [104], respectively. Both 2A-HA and 3A-HA viruses have replication kinetics similar to the wt and were propagated and quantified the same way as the wt virus.

### Plasmids and reagents

pCCTα-RFP was constructed by cloning the CCTα-coding sequence purchased from DNASU plasmid depository (clone ID HsCD00515560) into pmRFP-N1 vector (Clontech). Plasmids pTM-2A-3D, pTM-2B-3D, and pTM-2Amut-3D were described previously [26]. Plasmid pcDNA3-ACSL3-HA was generously provided by Dr. Joachim Füllekrug, University of Heidelberg, Germany. DNA transfections were performed with Mirus 2020 reagent according to manufacturer’s recommendation. Bodipy 500/510 C4/C9 (a fluorescent long chain fatty analog), Bodipy 493/503 (lipid droplets stain), Alexa-488 azide, and cell click chemistry kit were from Molecular Probes (Thermo Fisher Scientific). Cell culture media and supplements were from Thermo Fisher (GIBCO brand). Propargyl choline was synthesized as described in [58]. Digitonin was from Calbiochem. Triton-X100 was from Promega. Saponin, DEUP, Orlistat and bafilomycin were from Sigma Aldrich. Formaldehyde, glutaraldehyde, and cacodylate buffer were from Electron Microscopy Sciences. Recombinant universal type I interferon was from PBL Interferon Source.

### Antibodies

Mouse monoclonal anti-poliovirus 2C and 2B were described in [105]. Rabbit polyclonal anti-polio 3D antibodies were developed by Chemicon using recombinant 3D protein as immunogen. Rabbit monoclonal anti-CCTα, anti-eIF2α, anti-eIF2α (phospho Ser51), anti-IRF3, anti-IRF3 (phospho Ser396), anti-HSL, anti-ATGL, anti-STAT1, anti-Viperin, anti-ISG15 and anti-HA antibodies used in western blots and immunofluorescence were from Cell Signaling. Mouse monoclonal anti-dsRNA antibodies were from English and Scientific Consulting Kft. Mouse monoclonal anti-GBF1 antibodies were from BD Biosciences; rabbit anti-PI4KIIIβ were from EMD Millipore. Mouse monoclonal antibodies against Iκβ were a kind gift from Dr. John Patton (University of Maryland). Alexa dyes conjugated antibodies were from Molecular Probes (Thermo Fisher); secondary HRP-conjugated antibodies were from Amersham. Mouse monoclonal anti-HA antibody used for co-IP was from Santa Cruz Biotechnology.

### Co-immunoprecipitation assay

Co-IP was performed using Classic IP/Co-IP kit (Pierce) according to the manufacturer’s protocol. Briefly, HeLa cells grown on six well plate were infected with 2A-HA or 3A-HA polioviruses at an MOI of 10 PFU/cell. Control cells were transfected with a plasmid expressing ACSL3-HA, or infected with a wt poliovirus at an MOI of 10 PFU/cell. At 6 h p.i. (~24 h post transfection with the ACSL3-HA expressing plasmid), the cells were harvested in 750 μl of IP lysis buffer supplemented with a Protease Inhibitor Cocktail (Sigma-Aldrich‎). Lysates were clarified by low speed centrifugation and protein concentration was determined using Bradford reagent (Bio-Rad). The amount of lysates corresponding to 1 mg of total protein was mixed with 4 μg of mouse monoclonal anti-HA antibody (Santa Cruz Biotechnology) in a total volume of 500 μl of IP lysis buffer and incubated with rotation during 2 hours at room temperature. Then, pre-washed protein A/G magnetic beads from the kit were added, and the samples were incubated with rotation for one more hour. The beads were collected with a magnetic stand and washed three times with IP lysis buffer. Bound proteins were eluted with elution buffer provided in the kit.

### Long chain FA incorporation assay

Metabolic targeting of long chain FA was monitored using Bodily 500/510 C4/C9, a C18 backbone long chain FA analog with incorporated fluorescent group essentially as described in [106]. Briefly, the cells were incubated in medium supplemented with 0.4 μM of the fluorescent FA analog for 1 h either pre-infection, or at the indicated times post infection or post transfection. The cells were fixed with 4% formaldehyde in PBS and processed for microscopy observations or quantitation of the fluorescent signal using Tecan Infinite M1000 plate reader.

### Propargylcholine incorporation assay

For labeling of newly-synthesized phospholipids the cells were incubated in balanced Earle solution supplemented with 100 μM of propargylcholine for 1 hour at the indicated time p.i. Immediately after the incubation with propargylcholine, the cells were fixed with 4% formaldehyde in PBS for 20min, washed with PBS for 3 times and processed for click-chemistry labeling with Alexa 488 azide using Click-it cell reaction buffer kit. Florescence was quantified with Tecan Infinite M1000 plate reader.

### Lipid droplets staining

The cells were incubated with 5 μM of Bodipy 493/503 (lipid droplets stain) for 15 min in PBS. 5 mM stock solution of Bodipy 493/503 was prepared in DMSO.

### Digitonin permeabilization assay

HeLa cells were grown on 12-well plate and were incubated after poliovirus infection for the indicated periods p.i. For permeabilization, the cells were washed once with KHM buffer (110 mM K-acetate, 2 mM MgCl_2_, 20 mM HEPES-KOH, pH 7.4) and incubated for 5 min in 50 μg/ml fresh digitonin solution in KHM (KHM buffer without digitonin for control cells) at room temperature. After permeabilization, the cells were washed twice with KHM and lysed with mild lysis buffer (0.1 M Tris-HCl pH 7.8; 0.5% Triton-×100) supplemented with protease inhibitors cocktail (Sigma-Aldrich). The lysate cleared by low-speed centrifugation was used for western blot analysis.

### siRNA

Previously validated siRNA targeting human CCTα (GGCUUCACGGUGAUGAACG) and control non-targeting siControl siRNA were from Dharmacon. HeLa cells were plated at 10000 cells/well in a 96 well plate and transfected with siRNA with Dharmafect 1 transfection reagent (Dharmacon) according to manufacturer’s recommendations. After 72 hours of incubation with siRNA, the cells were infected with poliovirus and assessed for activation of membrane synthesis using incorporation of the fluorescent long chain FA analog Bodily 500/510 C4/C9.

### Expression of viral proteins using vaccinia-T7 system

Purified recombinant vaccinia virus expressing T7 RNA polymerase (VT7-3 [36]) was a gift from Dr. Ioannis Bossis, University of Maryland. HeLa cells were transfected with pTM- based plasmids coding for fragments of polio cDNA under transcriptional control of T7 RNA polymerase promoter and translational control of EMCV IRES respectively, with Mirus 2020 DNA transfection reagent and simultaneously infected with 10 PFU/cell of the vaccinia-T7 virus. The next day, cells grown on glass cover-slips were fixed with 4% formaldehyde in PBS and processed for microscopy analysis.

### qPCR assay profiling of gene expression

The assessment of expression of 84 genes involved in the cellular anti-viral response was performed using RT^2^ Profiler PCR Array (Qiagen) according to manufacturer’s recommendations. Briefly, cellular mRNA was isolated using RNAeasy kit (Qiagen) and cDNA was synthesized using RT^2^ First Strand Kit (Qiagen). Quality of the isolated RNA and the lack of genomic DNA contamination was confirmed using RT^2^ RNA QC PCR Array (Qiagen). qPCR data were normalized to average data of housekeeping gene transcripts (beta actin, glyceraldehyde-3-phosphate dehydrogenase (GAPDH), and hypoxanthine phosphoribosyltransferase 1), and analyzed using the ΔΔCt method.

### Immunofluorescence

The cells grown on coverslips in 12 well plates were fixed with 4% formaldehyde in PBS for 20 min and washed for 3 times with PBS. For regular permeabilization assays, the cells were incubated for 5 min in 0.2% Triton X100 in PBS followed by 1 h incubation in 3% membrane blocking agent (Amersham) in PBS. The same blocking solution was used for dilution of primary and secondary antibodies. For mild permeabilization assay primary and secondary antibodies were diluted in 0.02% saponin in PBS containing 5% fetal bovine serum as a blocking agent. The cells were incubated with all antibodies for one hour. Processed coverslips were mounted on slides using Fluoromount-G medium (Electron Microscopy Sciences). Images were taken using either Zeiss Axiovert 200M fluorescent or LSM 510 confocal microscope.

### Electron microscopy

Cells grown in 12 well plates on glass coverslip were fixed with 2.5% glutaraldehyde /4% paraformaldehyde in 0.1 M sodium cacodylate buffer and processed for transmission EM imaging at the University of Maryland School of Medicine core facility.

### Image processing and statistical analysis

Digital images were processed using Adobe Photoshop software. All changes were applied equally to the whole image, and the same parameters were applied to images from the same experiment. The number of lipid droplets per cell was calculated using Fiji distribution of ImageJ software (NIH), with Analyze Particle module. Western blot signals were quantified using Image Studio software (Li-Cor). For statistical calculations, at least 100 cells from different fields, or 3 western blot membranes from independent experiments were analyzed for each data point. Statistical significance was calculated by two-tailed unpaired t-test using GraphPad Prizm software package.

## Supporting information

S1 Fig **A.** Replication of poliovirus is not affected by inhibitors of fatty acid synthase (orlistat); lipid droplet-associated lipases (DEUP) and lysosome acidification (bafilomycin). HeLa cells were infected with 50 PFU/cell of poliovirus and incubated for 4 h in the presence of 10μM orlistat, 400μM DEUP or 2μM bafilomycin. Expression of the viral non-structural protein 2C is shown, actin is shown as a loading control. **B.** Lypophagy is not required for activation of PC synthesis upon infection. HeLa cells were infected with poliovirus at an MOI of 10 PFU/cell, and were incubated with 2μM of bafilomycin. At 5 h p.i., the incubation medium was replaced with fresh pre-warmed balanced Earle solution containing propargylcholine. The cells were fixed at 6 h p.i. and processed for click-chemistry-based detection of incorporated propargylcholine and staining of nuclear DNA with Hoechst 33332 for normalization. Propargylcholine incorporation was normalized to that in mock-infected cells. **C.** Non-significant variability of poliovirus replication in independent choline deprivation experiments. HeLa cells pre-incubated in choline-free medium for ~72h were infected with poliovirus and were incubated after infection either in choline-free or choline-supplemented medium. Expression of the viral non-structural protein 2C is shown. The right panel shows viral replication in the experiment used for EM images presented on Fig 7.
(PDF)

S2 Fig **A.** No significant recruitment of MGL to lipid droplets in either infected or mock-infected HeLa cells. HeLa cells were infected (mock-infected) with poliovirus at an MOI of 10 PFU/cell and at 4 h p.i., they were fixed and processed for immunofluorescent analysis of MGL. **B.** Recruitment of ATGL to lipid droplets early during poliovirus replication cycle. HeLa cells were infected (mock-infected) with poliovirus at an MOI of 10 PFU/cell and at 3 h p.i., they were fixed and processed for immunofluorescent analysis of a viral antigen 2B and ATGL. Arrows indicate recruitment of ATGL to lipid droplets.
(PDF)

S3 Fig Translocation of GBF1 and PI4KIIIβ does not depend on membrane synthesis. HeLa cells pre-incubated in choline-free medium for ~72h were infected with poliovirus at an MOI of 10 PFU/cell and were incubated after infection either in choline-free or choline-supplemented medium for 4 h. GBF1 and PI4KIIIβ are concentrated in the Golgi area of mock-infected cells and translocate to perinuclear ring-like structures upon infection in cells incubated in either cholen-free or choline-supplemented media. Note the normal morphology of mock-infected cells incubated for ~78h in choline-free medium.
(PDF)

S4 Fig Inhibition of hydrolysis of lipids in lipid droplets affects the development of poliovirus replication organelles. HeLa cells were infected with 10 PFU/cell of poliovirus and incubated with 400μM of DEUP for 4 h p.i. **A.** Transmission EM image, arrows indicated scattered clusters of replication organelles in DEUP-treated cells. **B.** Distribution of the viral antigen 2B visualized in DEUP-treated and control cells after Triton X-100 permeabilization.
(PDF)

S5 Fig **A.** Degradation of IκB in infected cells does not depend on activation of membrane synthesis. HeLa cells were pre-incubated in choline-free medium for ~72h and were infected with poliovirus at an MOI of 10 PFU/cell and incubated in either a choline-free- or a choline-supplemented medium for 6 h. **B.** Differential expression of anti-viral response genes in choline-deprived and choline-supplemented poliovirus-infected cells. HeLa cells were pre-incubated in choline-free medium for ~72h and were infected with poliovirus at an MOI of 10 PFU/cell and incubated in either a choline-free- or a choline-supplemented medium after infection. At 6 h p.i., the cellular RNA was isolated and analyzed with a qPCR panel profiling 84 human genes involved in anti-viral response (Qiagen). The genes whose expression demonstrated statistically significant difference in expression more than 1.5x are shown. IL6, interleukin 6 (GenBank ID: NM_000600), a cytokine involved in inflammation and the maturation of B cells [107]. NFKBIA, NFKB inhibitor alpha (GenBank ID: NM_020529), encodes a member of the NF-kappa-B inhibitor family which is involved in the control of inflammation [108]. JUN, Jun proto-oncogene, AP-1 transcription factor subunit (GenBank ID: NM_002228), involved in the TLR signaling and control of inflammation [108]. CYLD, CYLD lysine 63 deubiquitinase, (GenBank ID: NM_015247), a negative regulator of multiple signaling pathways [109]. FOS, Fos proto-oncogene, AP-1 transcription factor subunit; subunit (GenBank ID: NM_005252), involved in the TLR signaling and control of inflammation [108]. IL8, interleukin 8 (GenBank ID: NM_000584), a major mediator of the inflammatory response [110]. **C.** Interferon-stimulated genes are expressed similarly in non-infected cells in choline-free and choline-supplemented media. HeLa cells were incubated for 60 h without choline and then incubated overnight with 20 units of universal type 1 interferon also in choline-free medium. After that the IFN-containing medium was removed and the cells were incubated in either choline-free or choline-supplemented medium for additional 6 or 24h.
(PDF)

S6 Fig A list of genes involved in the anti-viral response whose expression was reliably detected in choline-deprived and choline-supplemented poliovirus-infected cells in a representative experiment. HeLa cells were pre-incubated in choline-free medium for ~72 h and were infected with poliovirus at an MOI of 10 PFU/cell and incubated in either choline-free- or choline-supplemented medium after infection. At 6 h p.i., the cellular RNA was isolated and analyzed with a qPCR panel profiling 84 human genes involved in anti-viral response (Qiagen.
(XLSX)
