# Supplementary figures and images for: Phospholipid synthesis fueled by lipid droplets drives the structural development of poliovirus replication organelles
Source: PLoS Pathog. 2018 Aug 27;14(8):e1007280. doi: 10.1371/journal.ppat.1007280 (PMC6128640; doi:10.1371/journal.ppat.1007280)

**A**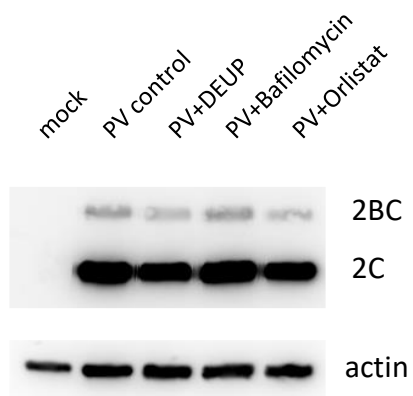**B**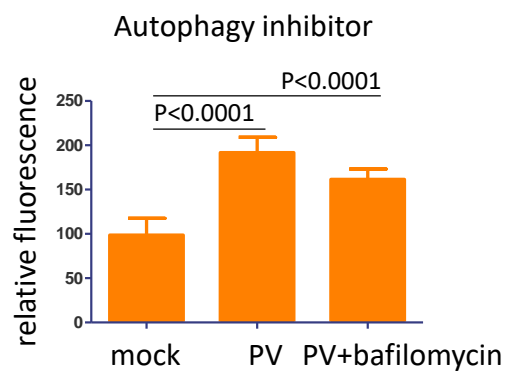**C**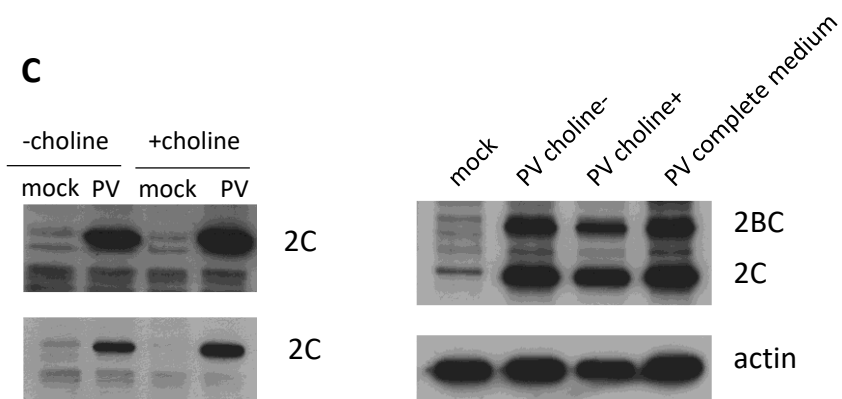

Supplementary figure 1

Supplement: S1 Fig — A. Replication of poliovirus is not affected by inhibitors of fatty acid synthase (orlistat); lipid droplet-associated lipases (DEUP) and lysosome acidification (bafilomycin). HeLa cells were infected with 50 PFU/cell of poliovirus and incubated for 4 h in the presence of 10μM orlistat, 400μM DEUP or 2μM bafilomycin. Expression of the viral non-structural protein 2C is shown, actin is shown as a loading control. B. Lypophagy is not required for activation of PC synthesis upon infection. HeLa cells were infected with poliovirus at an MOI of 10 PFU/cell, and were incubated with 2μM of bafilomycin. At 5 h p.i., the incubation medium was replaced with fresh pre-warmed balanced Earle solution containing propargylcholine. The cells were fixed at 6 h p.i. and processed for click-chemistry-based detection of incorporated propargylcholine and staining of nuclear DNA with Hoechst 33332 for normalization. Propargylcholine incorporation was normalized to that in mock-infected cells. C. Non-significant variability of poliovirus replication in independent choline deprivation experiments. HeLa cells pre-incubated in choline-free medium for ~72h were infected with poliovirus and were incubated after infection either in choline-free or choline-supplemented medium. Expression of the viral non-structural protein 2C is shown. The right panel shows viral replication in the experiment used for EM images presented on Fig 7. (PDF) [file ppat.1007280.s001.pdf]

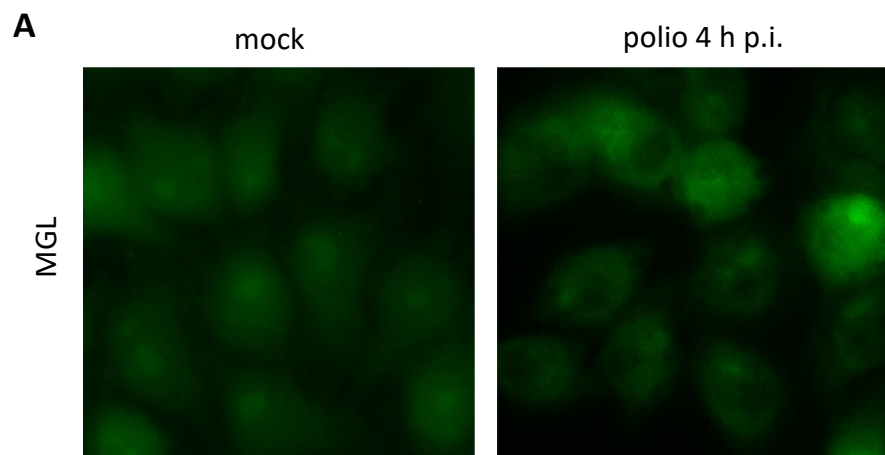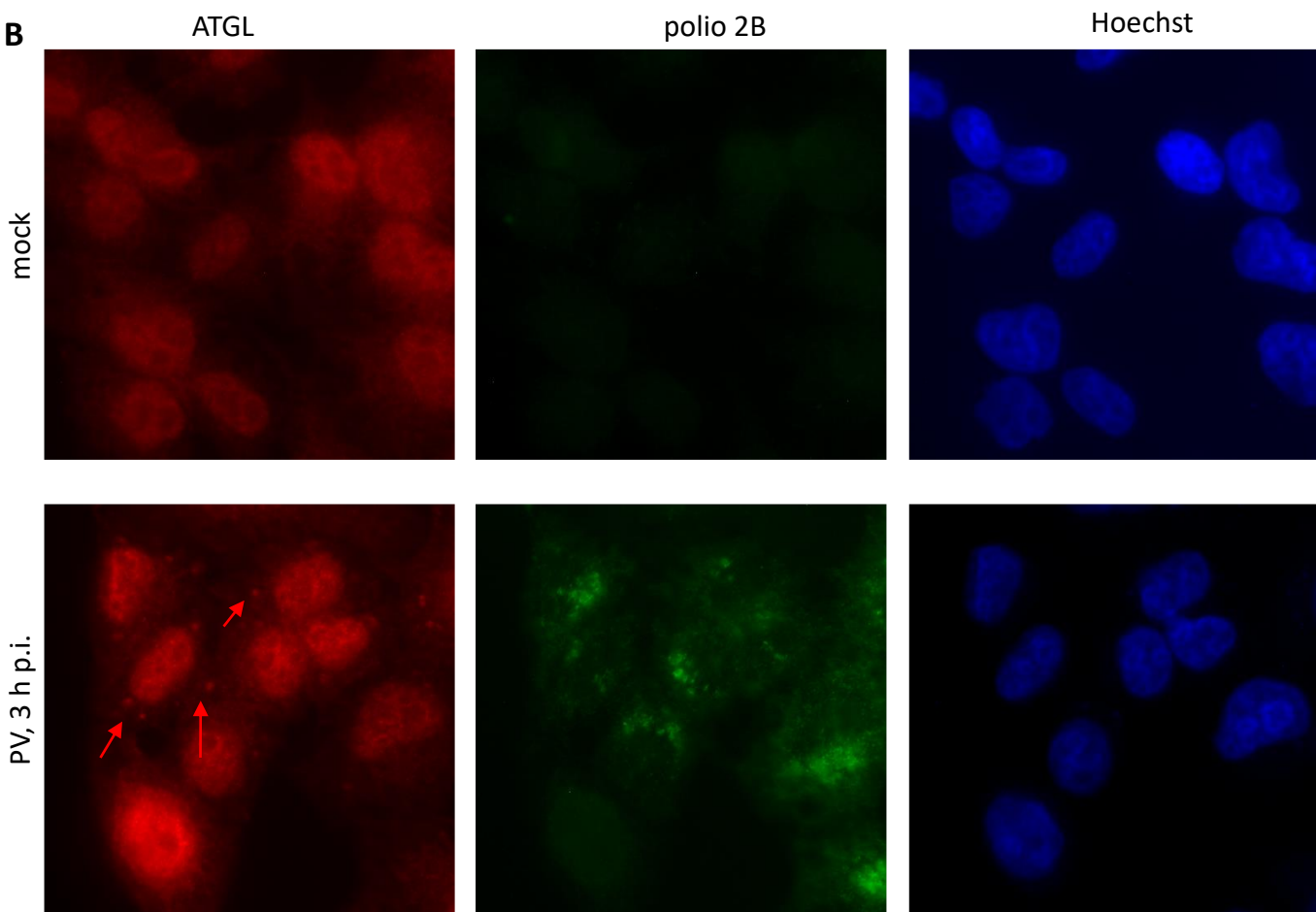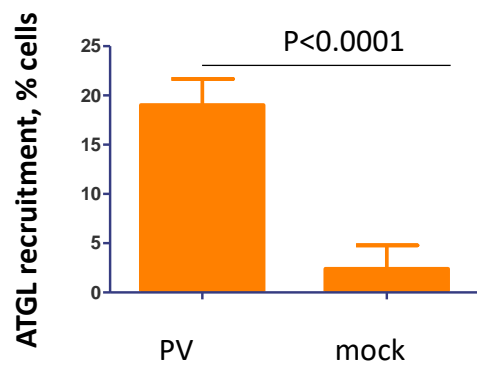

Supplementary figure 2

Supplement: S2 Fig — A. No significant recruitment of MGL to lipid droplets in either infected or mock-infected HeLa cells. HeLa cells were infected (mock-infected) with poliovirus at an MOI of 10 PFU/cell and at 4 h p.i., they were fixed and processed for immunofluorescent analysis of MGL. B. Recruitment of ATGL to lipid droplets early during poliovirus replication cycle. HeLa cells were infected (mock-infected) with poliovirus at an MOI of 10 PFU/cell and at 3 h p.i., they were fixed and processed for immunofluorescent analysis of a viral antigen 2B and ATGL. Arrows indicate recruitment of ATGL to lipid droplets. (PDF) [file ppat.1007280.s002.pdf]

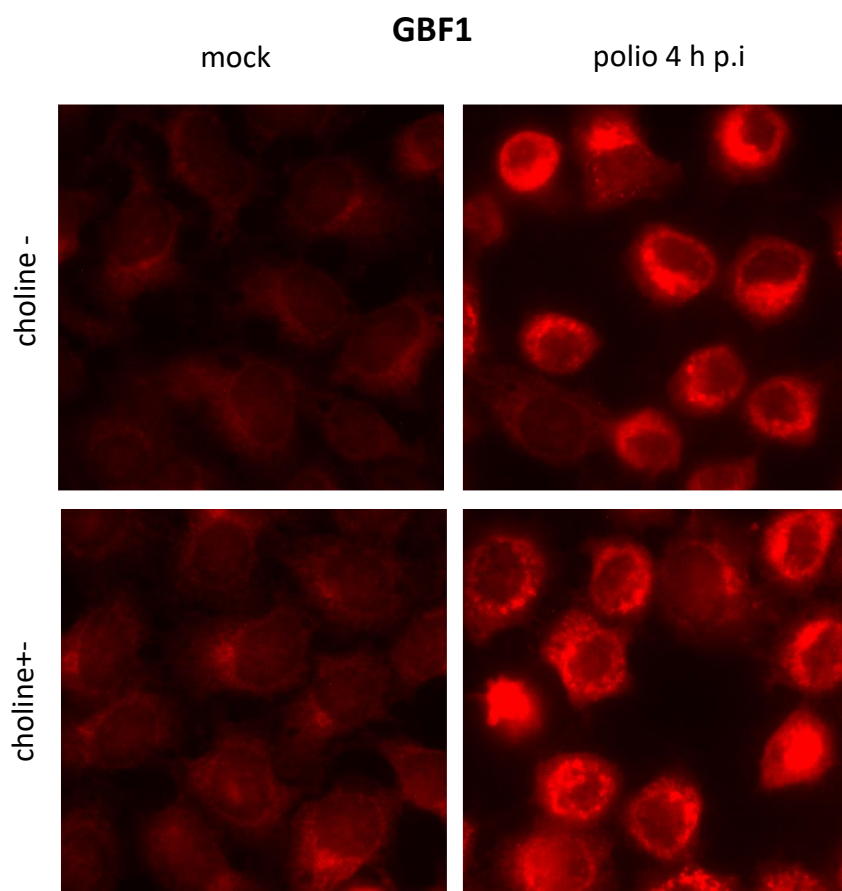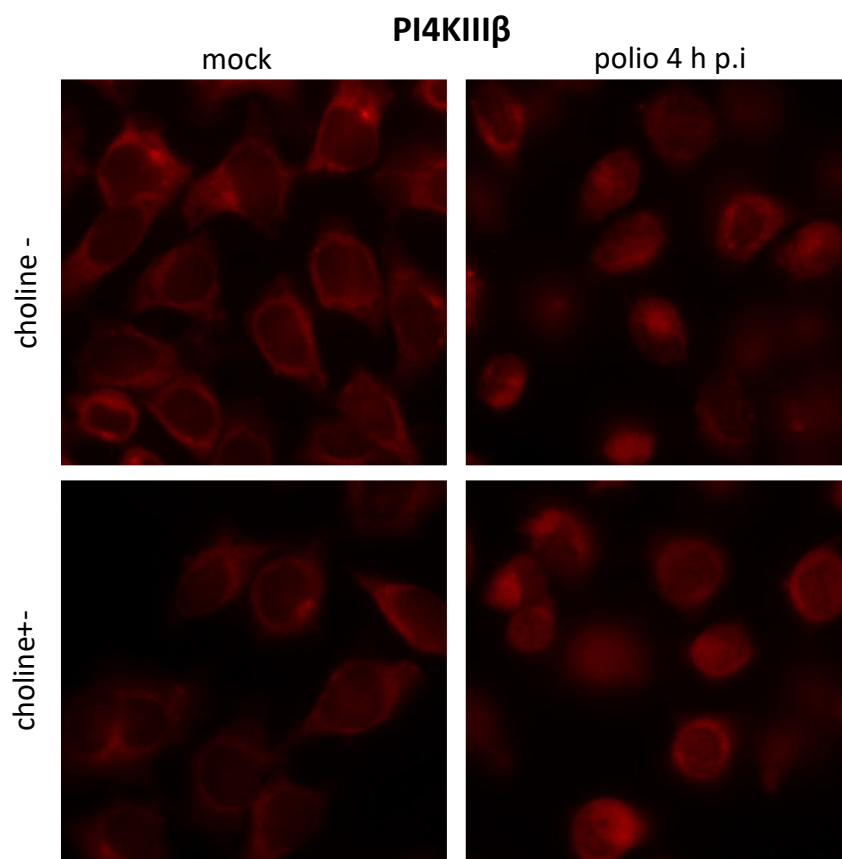

Supplementary figure 3

Supplement: S3 Fig — HeLa cells pre-incubated in choline-free medium for ~72h were infected with poliovirus at an MOI of 10 PFU/cell and were incubated after infection either in choline-free or choline-supplemented medium for 4 h. GBF1 and PI4KIIIβ are concentrated in the Golgi area of mock-infected cells and translocate to perinuclear ring-like structures upon infection in cells incubated in either cholen-free or choline-supplemented media. Note the normal morphology of mock-infected cells incubated for ~78h in choline-free medium. (PDF) [file ppat.1007280.s003.pdf]

A

control

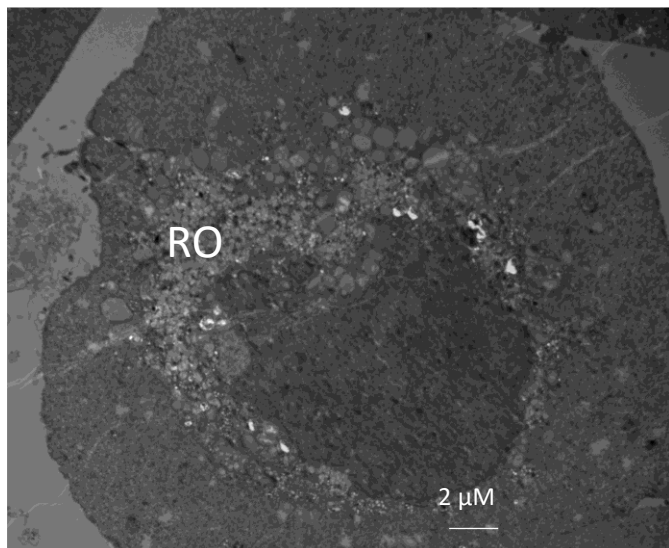

DEUP

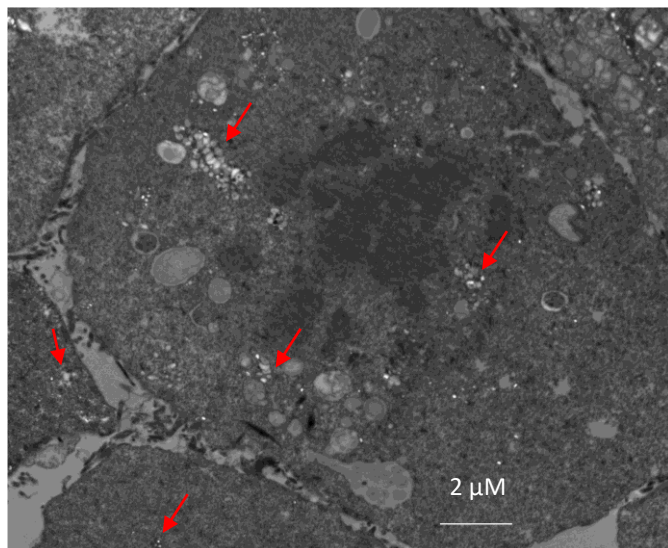

B

control

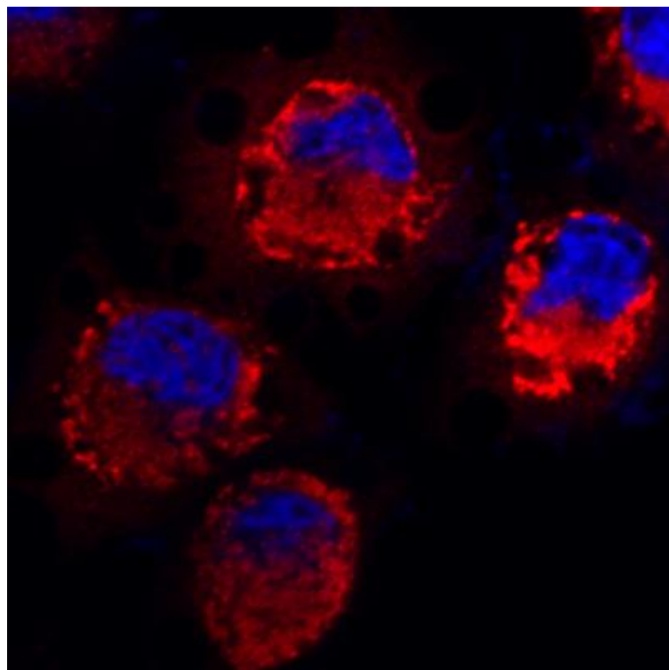

DEUP

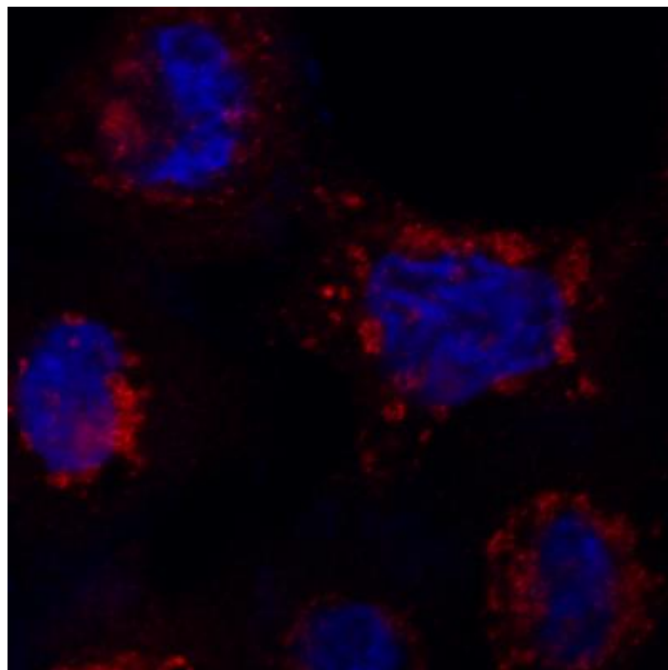

Supplementary figure 4

Supplement: S4 Fig — HeLa cells were infected with 10 PFU/cell of poliovirus and incubated with 400μM of DEUP for 4 h p.i. A. Transmission EM image, arrows indicated scattered clusters of replication organelles in DEUP-treated cells. B. Distribution of the viral antigen 2B visualized in DEUP-treated and control cells after Triton X-100 permeabilization. (PDF) [file ppat.1007280.s004.pdf]
