# Supplementary material for: Phospholipid synthesis fueled by lipid droplets drives the structural development of poliovirus replication organelles
Source: PLoS Pathog. 2018 Aug 27;14(8):e1007280. doi: 10.1371/journal.ppat.1007280 (PMC6128640; doi:10.1371/journal.ppat.1007280)
Supplement: S5 Fig — A. Degradation of IκB in infected cells does not depend on activation of membrane synthesis. HeLa cells were pre-incubated in choline-free medium for ~72h and were infected with poliovirus at an MOI of 10 PFU/cell and incubated in either a choline-free- or a choline-supplemented medium for 6 h. B. Differential expression of anti-viral response genes in choline-deprived and choline-supplemented poliovirus-infected cells. HeLa cells were pre-incubated in choline-free medium for ~72h and were infected with poliovirus at an MOI of 10 PFU/cell and incubated in either a choline-free- or a choline-supplemented medium after infection. At 6 h p.i., the cellular RNA was isolated and analyzed with a qPCR panel profiling 84 human genes involved in anti-viral response (Qiagen). The genes whose expression demonstrated statistically significant difference in expression more than 1.5x are shown. IL6, interleukin 6 (GenBank ID: NM_000600), a cytokine involved in inflammation and the maturation of B cells [107]. NFKBIA, NFKB inhibitor alpha (GenBank ID: NM_020529), encodes a member of the NF-kappa-B inhibitor family which is involved in the control of inflammation [108]. JUN, Jun proto-oncogene, AP-1 transcription factor subunit (GenBank ID: NM_002228), involved in the TLR signaling and control of inflammation [108]. CYLD, CYLD lysine 63 deubiquitinase, (GenBank ID: NM_015247), a negative regulator of multiple signaling pathways [109]. FOS, Fos proto-oncogene, AP-1 transcription factor subunit; subunit (GenBank ID: NM_005252), involved in the TLR signaling and control of inflammation [108]. IL8, interleukin 8 (GenBank ID: NM_000584), a major mediator of the inflammatory response [110]. C. Interferon-stimulated genes are expressed similarly in non-infected cells in choline-free and choline-supplemented media. HeLa cells were incubated for 60 h without choline and then incubated overnight with 20 units of universal type 1 interferon also in choline-free medium. After that the IFN-conta [file ppat.1007280.s005.pdf]

A

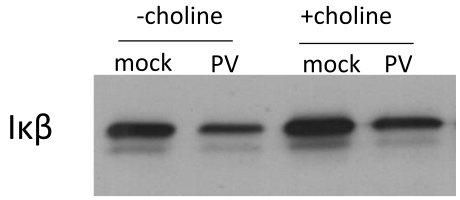

Degradation of IκB in infected cells does not depend on activation of membrane synthesis. HeLa cells were pre-incubated in choline-free medium for ~72h and were infected with poliovirus at an MOI of 10 PFU/cell and incubated in either a choline-free- or a choline-supplemented medium for 6 h.

B

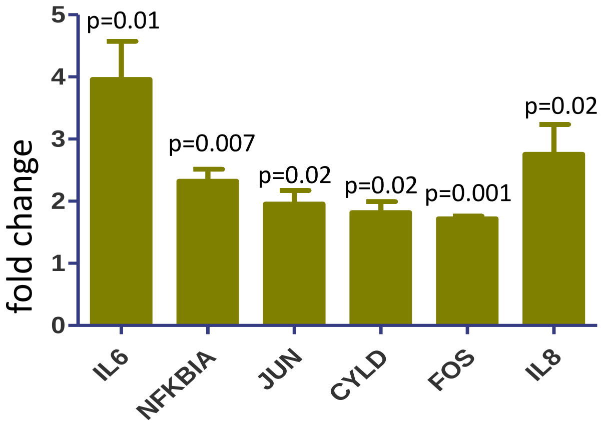

Genes with more than 1.5x activation of expression in infected cells with inhibited membrane synthesis. Results are from 2 independent experiments, p values (ch- vs ch+ for a corresponding sample) are shown.

C

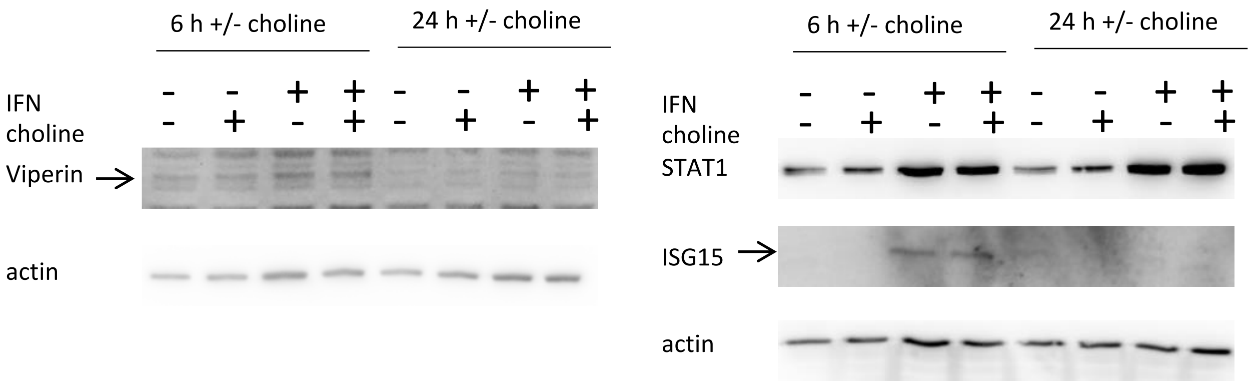

Interferon-stimulated genes are expressed similarly in non-infected cells in choline-free and choline-supplemented media. HeLa cells were incubated for 48 h without choline and then incubated overnight with 20 units of universal type 1 interferon also in choline-free medium. After that the IFN-containing medium was removed and the cells were incubated in either choline-free or choline-supplemented medium for additional 6 or 24h.

Supplementary figure 5
